# Hydrogen sulfide decreases high glucose/palmitate-induced autophagy in endothelial cells by the Nrf2-ROS-AMPK signaling pathway

**DOI:** 10.1186/s13578-016-0099-1

**Published:** 2016-05-23

**Authors:** Jiaqi Liu, Jichao Wu, Aili Sun, Yu Sun, Xiangjing Yu, Ning Liu, Shiyun Dong, Fan Yang, Linxue Zhang, Xin Zhong, Changqing Xu, Fanghao Lu, Weihua Zhang

**Affiliations:** Department of Pathophysiology, Harbin Medical University, Harbin, 150086 China

**Keywords:** Hydrogen sulfide, Type II diabetes, Vascular endothelial cells, Autophagy, Oxidative stress

## Abstract

**Background:**

Excessive autophagy induced by extravagant oxidative stress is the main reason for diabetes-induced vascular endothelial cells dysfunction. Hydrogen sulfide (H_2_S) has anti-oxidative effects but its regulation on excessive autophagy of vascular endothelial cells is unclear.

**Methods:**

In this study, aorta of db/db mice (28 weeks old) and rat aortic endothelial cells (RAECs) treated with 40 mM glucose and 500 μM palmitate acted as type II diabetic animal and cellular models, respectively, and 100 μMNaHS was used as an exogenous H_2_S donor. The apoptosis level was measured by terminal deoxynucleotidyl transferase mediated dUTP nick-end labeling (TUNEL) staining and Hoechst 33342/PI staining. The activities of SOD, CAT and respiratory complexes were also measured. The mRNA levels of SOD and CAT were detected by real-time PCR. AMPK-siRNA was used to detect the effect of AMPK on autophagy. Western blotting was used to detected the protein level.

**Results:**

H_2_S production was decreased (p < 0.05, p < 0.01) both in vitro and in vivo; NaHS treatment rescued this impairment (p < 0.05, p < 0.01). The expression of adhesive proteins was increased (p < 0.05, p < 0.01) both in vitro and in vivo; NaHS attenuated (p < 0.05, p < 0.01) these alterations. NaHS could protect endothelial cells against apoptosis induced by type II diabetes (p < 0.05, p < 0.01). Furthermore, the expressions and activities of SOD and CAT were impaired (p < 0.05, p < 0.01) in endothelial cells of diabetes II; NaHS treatment attenuated (p < 0.05) this impairment. NaHS also increased ATP production (p < 0.05) and activities of respiratory complexes (p < 0.05), and the ratio of p-AMPK to AMPK was also decreased by NaHS (p < 0.01). The level of autophagy in endothelial cells was also decreased (p < 0.05, p < 0.01) by NaHS treatment and AMPK-siRNA treatment. The expression of Nrf2 in the nuclei was increased (p < 0.05) by NaHS treatment.

**Conclusion:**

Exogenous H_2_S might protect arterial endothelial cells by suppressing excessive autophagy induced by oxidative stress through the Nrf2-ROS-AMPK signaling pathway.

**Electronic supplementary material:**

The online version of this article (doi:10.1186/s13578-016-0099-1) contains supplementary material, which is available to authorized users.

## Background

Diabetes mellitus is firmly established as a major threat to human health due to its severe complications in the cardiovascular system [1]. Endothelial cell dysfunction (ECD) plays an important role in these diabetes induced complications, which can result in the impairment of vasodilation, the barrier functions of endothelial cells, disturbances in proliferative capacities, impairment of the migratory and tube formation properties, impairment of angiogenic properties, attenuation of synthetic function, and deterrence of white blood cell adhesion and diapedesis [2]. Diabetes can be stratified into two groups, type I and type II, both of which can cause hyperglycemia, a major cause of ECD [3]. Type II diabetes can cause more risk factors for ECD, including insulin resistance [4] and obesity [5]. Therefore finding therapies that protect endothelial cells would create good prospects for treating diabetes.

It has been reported that increased reactive oxygen species (ROS) production in type I and type II diabetes contributes to ECD [6, 7]. One of the damage elements caused by ROS in endothelial cells is excessive autophagy or disruptive autophagy [8, 9]. In the physiological state, autophagy is a protective factor for endothelial cells, which can deliver cytoplasmic cargo to the lysosomes that scavenge waste within the cells [10]. In diabetes, the ROS is at a high level if hyperglycemia is not rectified. Autophagy will be interrupted, which can result in waste such as ubiquitin and injured organelles accumulating within endothelial cells [11], which are toxic to cells.

5-AMP-activated protein kinase (AMPK) plays a general role in coordinating growth and metabolism. It exists as an obligate heterotrimer, containing a catalytic subunit (α) and two regulatory subunits (β and γ). Under lowered intracellular ATP levels, AMP and ADP can directly bind to the regulatory subunits leading to a conformational change that activates phosphorylation of AMPK [12]. The role of AMPK in apoptosis is not clear, with both anti-apoptotic and pro-apoptotic actions being reported. In a number of cell types, including vascular smooth muscle cells, pancreatic cells and various cancerous cells [13, 14]. AMPK activation has been shown to be pro-apoptotic. This pro-apoptotic effect of AMPK has been attributed to inhibition of cell cycle progression, activation of the JNK pathway and caspase-3 activation, and up-regulation of the pro-apoptotic p53 protein [15]. AMPK can also trigger autophagy in a double-pronged mechanism of directly activating ULK1 and inhibiting the suppressive effect of mTORC1 complex I on ULK1 [16].

Hydrogen sulfide (H_2_S) is a newly found gas transmitter that has a strong anti-oxidative effect [17]. In mammalian tissues, the biosynthesis of H_2_S is catalyzed by the pyridoxal-5-phosphate-dependent enzymes, cystathionine-β-synthetase (CBS) and cystathionine-γ-lyase (CSE).In the cardiovascular system, H_2_S is mainly catalyzed by CSE. Studies have shown that the anti-oxidative effect of H_2_S is associated with the Keap1-Nrf2 signaling pathway [18]. Calvert initially reported that H_2_S promoted Nrf2 translocation to the nucleus to up-regulate the transcription of antioxidant genes [19], then Wang and colleagues showed that H_2_S sulfurates Keap1, which releases Nrf2 to the nucleus [20]. There is little evidence that illuminates the relation between H_2_S and excessive autophagy in endothelial cells. Therefore, we hypothesized that H_2_S can protect endothelial cells through inhibiting excessive autophagy in diabetes.

## Methods

### Animals

Ten-week-old diabetic mice (db/db mice) weighing 45–55 g were purchased from the Animal Model Institute of Nanjing. The animals were housed under diurnal lighting conditions and fed standard mouse chow and water throughout the study period. All animal experiments were performed in accordance with the Guide for the Care and Use of Laboratory Animals published by the China National Institutes of Health and approved by the Animal Care Committees of Harbin Medical University, China.

### Experimental groups

The animal experiment was divided into three groups. Each group included eight mice (n = 8). The db/db mice were divided into db/db and db/db with NaHS treatment. NaHS (100 μmol/kg) was given by intraperitoneal injection in the NaHS treatment group every 2 days.

### Cell culture and treatment

Primary cultures of rat aortic endothelial cells (RAECs), purchased from Chinese Academy of Sciences Cell Bank, were grown as monolayers at a density of 5 × 10^4^ cells/cm^2^ in Dulbecco’s modified Eagle medium (DMEM) and incubated at 37 °C in humidified air containing 5 % CO_2_. The medium contained 10 % calf serum, 100 units/ml penicillin and 100 μg/ml streptomycin. Two days after seeding, the cultured RAEC were randomly divided into the following six groups and treatments: control group (low glucose, LG, 5.5 mmol/L), high glucose (HG, 40 mM) + Palmitate (Pal, 500 μM), HG + Pal + NaHS (100 μM), HG + Pal + NAC (10 mM, an inhibitor of reactive oxygen species), HG + Pal +3-Methyladenine (3-MA, 5 mM, an inhibitor of autophagy) and HG + Pal + compound C (20 μM, an inhibitor of AMPK). Drugs were added directly to the culture for 48 h.

### Total protein extraction from diabetes mouse aortic and rat aortic endothelial cells Western blotting analysis

Diabetic mouse aortic and rat aortic endothelial cells were homogenized in 0.5 ml of RIPA buffer prior to being transferred into small tubes and rotated for 1 h at 4 °C. Solubilized proteins were collected after centrifugation at 12,000*g* for 30 min. The supernatant was collected and stored at −80 °C. The protein concentration of each sample was quantified by using the BCA protein assay kit. Polyacrylamide gels (12 %) were used for protein testing and equal amounts of proteins were applied, then electrotransferred onto a PVDF membrane (Millipore). The nonspecific proteins on membranes were blocked with 5 % non-fat dried milk for 2 h at room temperature. Membranes were incubated with 2 μg/mL rabbit anti-rat CSE (42 kDa, 1:1000), vWF (80 kDa, 1:1000), ITGβ1 (130 kDa, 1:1000), GP1βA (88 kDa, 1:1000), β-actin (42 kDa, 1:1000), Bcl-2 (26 kDa, 1:1000), Bax (21 kDa, 1:1000), beclin-1 (60 kDa, 1:1000), Atg7 (78 kDa, 1:1000), p62 (68 kDa, 1:1000), Lamp2 (50 kDa, 1:1000), Nrf2 (100 kDa, 1:1000) and P-AMPK/AMPK (62 kDa, 1:1000), mouse anti-rat LC3I/II (14–16 kDa, 1:1000) and rabbit anti-human Cleaved-caspase-3 (17 kDa, 1:1000), caspase-3 (32 kDa, 1:1000) polyclonal antibodies, overnight at 4 °C, respectively. All antibodies were from Protein tech Group, Inc., USA. Membranes were washed and then incubated with anti-mouse/anti-rabbit IgG antibody at a 1:1000 dilution for 1 h at room temperature. The specific complex was visualized using ECL plus Western blotting detection system. The relative intensities of protein bands were quantified by using a Bio-Rad Chemi EQ densitometer and Bio-Rad Quantity One software (Bio-Rad laboratories, Hercules, USA).

### Measurement of H_2_S in aorta of db/db mice

H_2_S content was measured on at least four samples from each group using the H_2_S content kit from Sigma-Aldrich (RAD171, USA) according to the manufacturer’s instructions. Absorbance at 670 nm was measured using a spectrophotometer from SAPE (Shanghai, China). H_2_S content was then calculated based on a standard curve of H_2_S solution.

### Detection of H_2_S in RAECs using H_2_S probe 7-Azido-4-Methylcoumarin

The turn-on fluorescence response of H_2_S in RAECs was tested using 7-Azido-4-Methylcoumarin, (C-7Az, Sigma), which has been proved to selectively respond to H_2_S. RAECs were incubated with 50 μM C-7Az PBS for 30 min, followed by washing of the cells with PBS. Visualization of the turn-on fluorescence response of C-7Az to H_2_S in RAECs was carried out using fluorescence microscopy with the excitation of a 720 nm laser. These results confirmed that excitation fluorescence imaging could be used to detect H_2_S through the triggered fluorescence response of C-7Az.

### Terminal deoxynucleotidyl transferase mediated dUTP nick-end labeling (TUNEL) staining

The TUNEL assay was used to test for apoptosis in RAECs. Cells from the control group, HG group and NaHS group were fixed in 4 % formalin overnight, dehydrated and then embedded in paraffin. Apoptotic cells were tested by a TdT DNA fragmentation detection kit (Roche, Mannheim, Germany), and the procedure was performed according to the kit’s protocol. The percentage of apoptotic cells was calculated as the ratio of the number of TUNEL-positive cells to the total number of cells.

### Hoechst 33342/PI staining for apoptosis assay

Cells were seeded and treated for 48 h in 24-well plates, washed three times with PBS, then incubated with 20 μg/ml Hoechst staining buffer for 15 min at 37 °C in the dark and then incubated with 20 μg/ml propidium iodide (PI) for 10 min. The percentage of apoptotic cells was observed using fluorescence microscopy.

### Analysis of mitochondrial transmembrane potential

Changes in mitochondrial transmembrane potential were assessed using the lipophilic cationic probe 5, 5′, 6, 6′-Tetrachloro-1, 1′, 3, 3′-tetraethyl-imida-carbocyanine iodide (JC-1). In brief, RAECs were seeded and treated for 48 h at 37 °C. After experimentation, cells were loaded with 2 μM JC-1 (Invitrogen) at 37 °C in the dark for 15 min and rinsed three times with cold PBS. Green fluorescence reflected the monomeric form of JC-1, and red fluorescence reflected the aggregate form. The cells were monitored using fluorescence microscopy.

### Immunofluorescence assays

For immunofluorescent staining with anti-LC3 antibody, the RAECs were fixed in 4 % paraformaldehyde for 30 min, then permeabilized with 0.5 % Triton X-100 for 30 min. Coverslips were blocked with goat serum and incubated for 1 h at 37 °C. Cells were incubated with anti-LC3 antibody at 4 °C overnight and washed three time with PBS, followed by incubation for 1 h with anti-rabbit IgG. Analysis and photomicrography were carried out with fluorescence microscopy.

### Monodansylcadaverine (MDC) assay for visualization of autophagic vacuoles

Cells were cultured and treated on cover slips 24 h later, cells were washed with PBS three times and fixed with a solution of 4 % paraformaldehyde for 30 min, then autophagic vacuoles were labeled with monodansylcadaverine (MDC) by incubating cells with 50 μM MDC in PBS at 37 °C for 30 min. Autophagic vacuoles analyzed by using fluorescence microscopy (Olympus, XSZ-D2).

### siRNA transfection

The RAECs (80 % confluent) were treated according to the manufacturer’s instructions with AMPK short interfering RNAs (siRNAs) (mouse; Santa Cruz Biotechnology) for 48 h to inhibit AMPK expression. Transfected cells were confirmed by Western blot analysis of AMPK protein expression.

### Mitochondrial ROS and cellular ROS level analysis

Mitochondrial ROS production was measured using Mito-SOX Red mitochondrial superoxide indicator (Invitrogen). RAECs were loaded with 5 μM Mito-SOX Red at 37 °C for 15 min. Red fluorescence was measured at 583 nm following excitation at 488 nm using a Zeiss LSM 510 inverted confocal microscope. Intracellular ROS levels were examined using the DCFH-DA staining method based on the conversion of non-fluorescent DCFH-DA to the highly fluorescent DCF upon intracellular oxidation by ROS. RAECs were seeded on coverslips and incubated (45 min, 37 °C, in the dark) in serum-free media containing DCFH-DA (10 μM/L) in the presence of control, high glucose and NaHS. After incubation, the conversion of DCFH-DA to the fluorescent product DCF was measured using a spectrofluorometer with excitation at 484 nm and emission at 530 nm. Background fluorescence (conversion of DCFH-DA in the absence of cells) was corrected by the inclusion of parallel blanks.

### Measurement SOD and CAT activities

RAECs were treated with high glucose and palmitate (500 μM), NaHS and NAC for 48 h, and then total protein was prepared. Superoxide dismutase (SOD) and catalase (CAT) in the supernatant were measured using a spectrophotometer (Jiancheng Institute of Bioengineering, Nanjing, China). All assays were conducted according to the kit instructions.

### Real-time RT-PCR

Total RNA and then cDNA were prepared from cells using TRIzol (Invitrogen) and the ReverAid™ First Strand cDNA Synthesis Kit (Fermentas, Lithuania). The primer sequences for Mn-SOD and CAT were forward 5′AGATGACTTGGGCAAGGTG3′; reverse 5′CAATCCCAATCACACCACAA3′ and forward 5′ACATGGTCTGGGACTTCTGG3′; and reverse 5′CCATTCGCATTAACCAGCTT3′. Expression levels were normalized to the house-keeping gene GAPDH (forward 5′-AGAAGGCTG GGGCTCATTTG-3′; reverse 5′-AGGGGCCATCCACAGTCTTC-3′). mRNA levels were acquired from the value of threshold cycle (*C*_t_) of the real-time PCR and normalized to GAPDH. Data were obtained from three separate experiments.

### Determination of ATP Levels

The level of ATP in RAECs was determined using the ATP Bioluminescence Assay Kit (Beyotime, China). Cultured cells were harvested from 6-well plates, then lysed with 200 μL lysis buffer from the ATP detection kit, followed by centrifugation at 12,000×*g* for 5 min at 4 °C. The luminescence from the 20 μL supernatant and standards was assayed in a luminometer together with 100 μL ATP detection buffer from the ATP detection kit. The standard curve of ATP concentration was prepared from a known amount (5 nM–10 μM).

### Mitochondrial enzyme complexes

Complex II–IV values were measured by using a spectrophotometer (GENMED, Shanghai, China). All assays were conducted according to the kit instructions.

### Statistical analysis

Data are expressed as the mean ± standard error (SEM). Statistical analysis was analyzed by one-way ANOVA. Differences between individual groups were analyzed using Student’s t test. P < 0.05 was considered statistically significant and p < 0.01 was considered very significant.

## Results

### Exogenous H_2_S improved H_2_S level in arteries of db/db mouse and RAECs

**Fig. 1:**
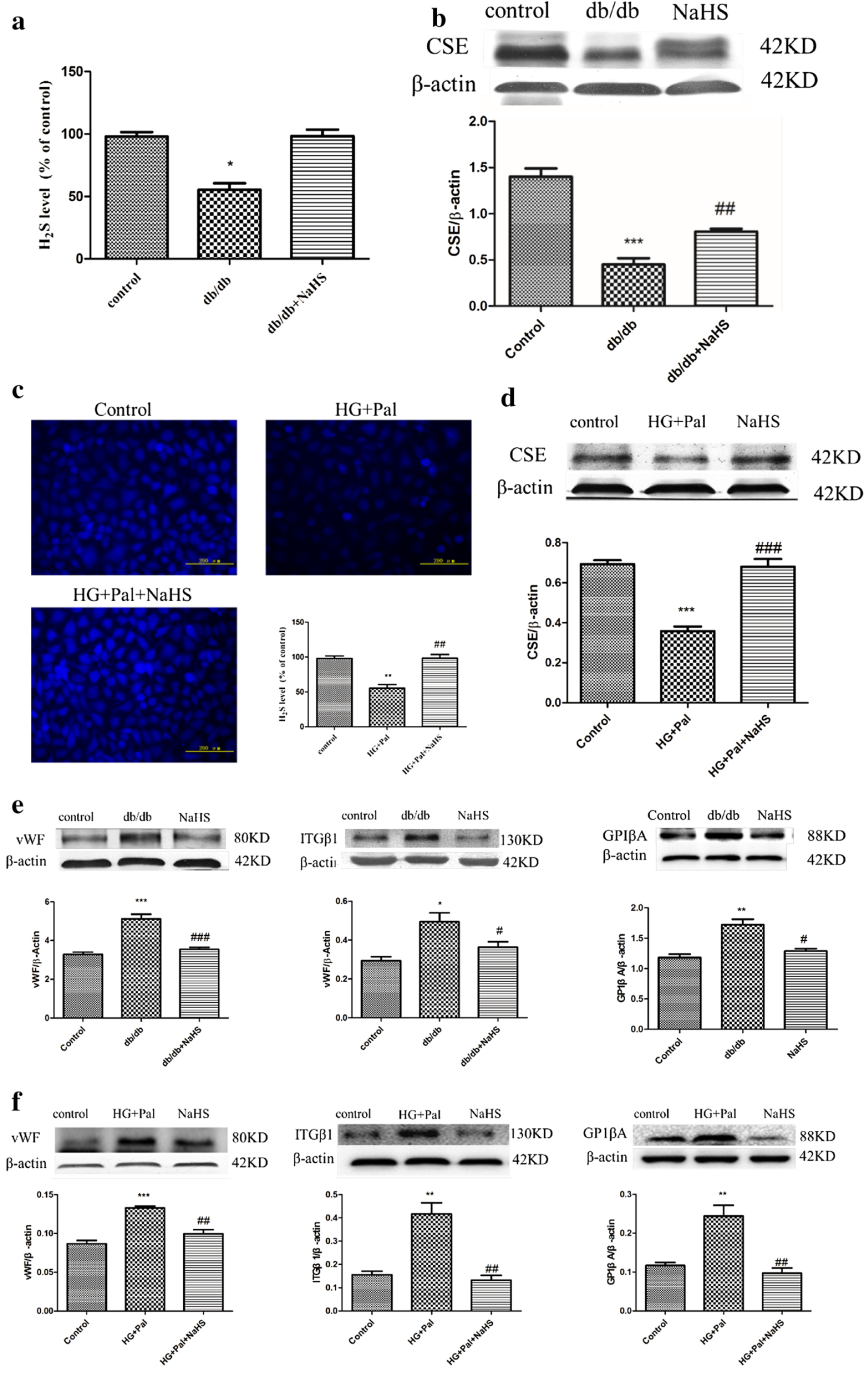
Exogenous H_2_S improved H_2_S level and adhesive function in arterial endothelial cells. **a** Eight–week-old db/db mice were treated by injection of 100 μmol/kg/day NaHS for 12 weeks. The arteries were selected to examine the H_2_S level. The results were expressed as the mean ± SD. (*p < 0.05 vs control group). **b** The CSE level of arteries was tested by Western blotting. (***p < 0.001 vs control group. ^##^p < 0.01 vs db/db mouse). **c** RAECs were treated with 40 mM glucose, 200 μM palmitate and 100 μM NaHS for 24 h, and the H_2_S level were detected. (**p < 0.01 vs control group. ^##^p < 0.01 vs HG + Pal group). **d** The CSE level of RAECs was tested by Western blotting. ***p < 0.001 vs control group. (^###^p < 0.001 vs HG + Pal group). **e** The expression of vWF, ITGβ1 and GP1βA was examined by Western blotting in vivo. **f** The expressions of vWF, ITGβ1 and GP1βA were examined by Western blotting in vitro (*p < 0.05 vs control group. **p < 0.01 vs control group. ^#^p < 0.05 vs db/db mouse. ^###^p < 0.001 vs db/db mouse. ^##^p < 0.01 vs HG + Pal group)

H_2_S is an important gaseous signal molecule in arteries, regulating the function of endothelial cells and smooth muscle cells. Firstly, the H_2_S levels and CSE expression, the H_2_S production enzyme, were tested in the aorta of db/db mice. The H_2_S level was significantly decreased in the arteries of db/db mice, and its level was recovered after NaHS injection (Fig. 1a). Meanwhile, the CSE expression that was down-regulated in the arteries of db/db mice was elevated after NaHS injection (Fig. 1b). Because the above tests illuminated the H_2_S production of arteries on the whole, we further tested whether exogenous H_2_S could affect RAECs. RAECs with the treatment of HG and palmitate were used to imitate the in vivo conditions. Our results revealed that the H_2_S level and CSE expression treated by HG and Pal were significantly decreased and that these alterations were attenuated after treatment by NaHS (Fig. 1c, d). These results suggested that the H_2_S production of arterial endothelial cells was altered in type II diabetes and the exogenous H_2_S could help recover these alterations.

### Exogenous H_2_S attenuated the adhesivity of vascular endothelial cells in db/db mice and RAECs

To further investigate the effects of H_2_S on arterial endothelial cell protection, we examined markers that could reflect the dysfunction of arterial endothelial cells, including the Von Willebrand factor (vWF), Integrin beta-1 (ITGβ1) and GP1βA. vWF is a surface membrane protein of endothelial cells, and it can bind with GP1βA, which mediates platelets adherence to endothelial cells [21]. ITGβ1 is capable of binding to intracellular adhesion molecule 1 (ICAM-1) forming the ItG-ICAM complex, which can facilitate erythrocyte adherence to endothelial cells [22]. These three factors are crucial to the hemostasis process and abnormal expression of which could facilitate thrombosis [23, 24]. Our results revealed that these markers were overexpressed both in vitro and in vivo of hyperglycemia and hyperlipidemia state, however, exogenous H_2_S attenuated these alterations (Fig. 1e, f).

### Exogenous H_2_S prevented apoptosis of RAECs

**Fig. 2:**
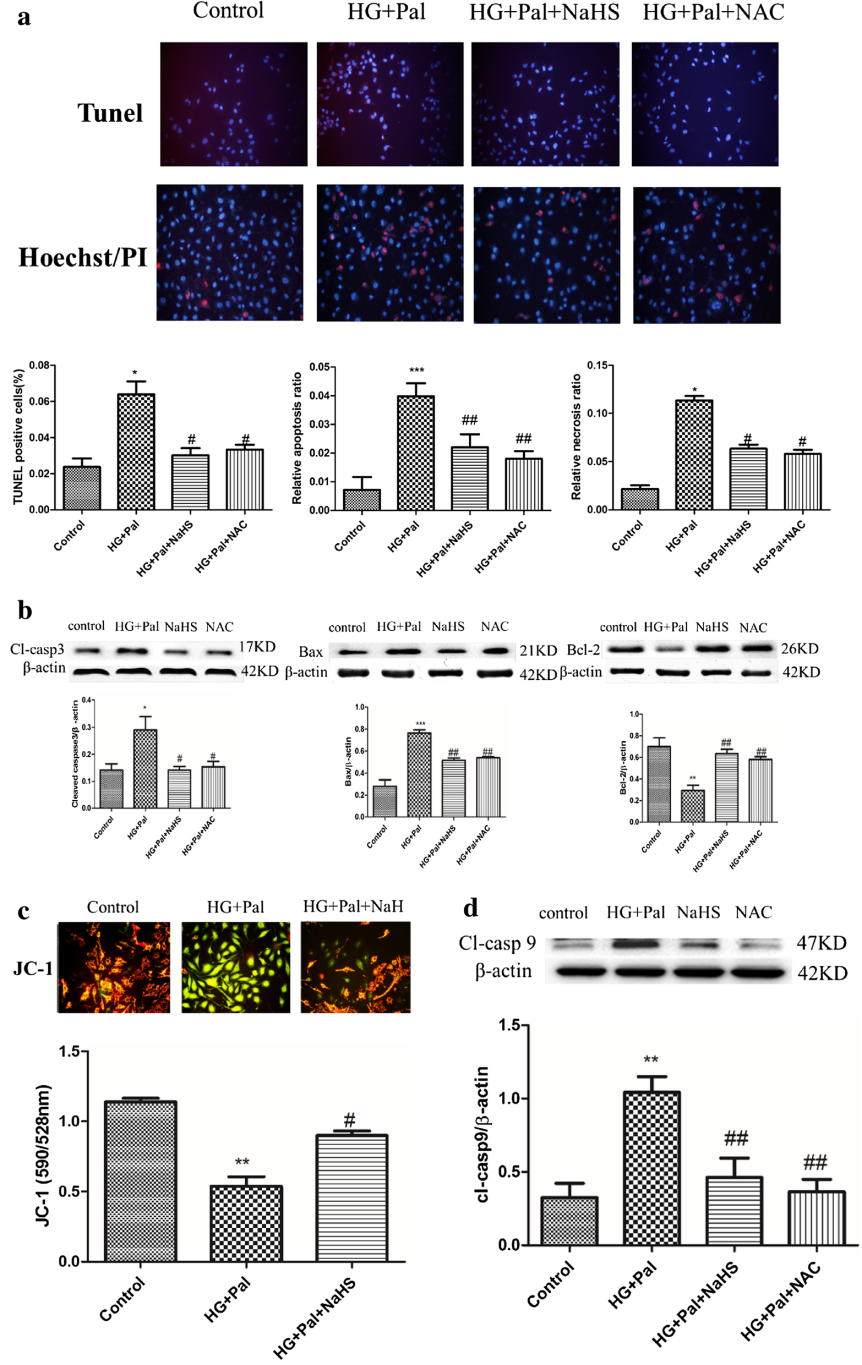
Exogenous H_2_S prevented apoptosis of RAECs. **a** RAECs were stained by TUNEL and Hoechst/PI. The apoptotic cells were stained in *red* in TUNEL assay. The nuclei of apoptotic cells showed condensate and necrotic cells were stained in *red* in Hoechst/PI assay. The quantity of positive cells was measured at least 200 cells in three different experiments. (***p < 0.001 vs control group. ^##^p < 0.01 vs HG + Pal group. ^###^p < 0.001 vs HG + Pal group). **b** The expression of cl-casp 3, Bax and Bcl-2 was tested by Western blotting. Each optical density (OD) value was standardized to that of β-actin. There data were summarized from at least three different experiments. (*p < 0.05 vs control group. **p < 0.01 vs control group. ***p < 0.001 vs control group. ^#^p < 0.05 vs HG + Pal group. ^##^p < 0.01 vs HG + Pal group). **c** JC-1 measured the mitochondrial membrane potential (ΔΨm). The effect of NaHS treatment on mitochondrial membrane potential in RAECs treated by HG + Pal. JC-1 spontaneously forms J-aggregates and exhibits red fluorescence (590 nm) under a high membrane potential. The JC-1 monomeric form shows green fluorescence (528 nm) under a low potential. The *red fluorescence* to *green fluorescence* ratio was measured. (**p < 0.01 vs control group. ^#^p < 0.01 vs HG + Pal group). **d** The expression of cl-casp 9 was examined by Western blotting. The OD value was standardized to that of β-actin. There data were summarized from at least three different experiments. (**p < 0.01 vs control group. ^##^p < 0.01 vs HG + Pal group)

Hyperglycemia and hyperlipidemia are characteristics of type II diabetes that can induce apoptosis in endothelial cells, so a protective effect should also be reflected in preventing apoptosis. Firstly, we detected NaHS pre-administration endothelial cells by time- and dose-dependent ways. Our result showed that 100 μM NaHS for 48 h significantly decreased endothelial cell death by Hoechst/PI staining (Additional file 1: Figure S1). And then we examined apoptotic cells using TUNEL and a Hoechst/PI assay to investigate whether NaHS treatment could attenuate endothelial cell apoptosis induced by HG and palmitate. Our results showed that the number of TUNEL-positive cells in the HG + Pal group was significantly increased compared with the control group. NaHS treatment markedly attenuated the apoptotic rate (Fig. 2a). Moreover, apoptotic markers including the cleavage of caspase 3 (cl-casp 3), and Bax and Bcl-2 were tested by Western blotting. Our results revealed that these markers were altered by HG and palmitate, which could lead to apoptosis in endothelial cells. These alterations were ameliorated by NaHS treatment (Fig. 2b). Hyperglycemia and hyperlipidemia can also induce mitochondrial injury that is characterized by changes in mitochondrial membrane potential. A JC-1 assay was used to examine mitochondrial membrane potential. As shown in the results, the mitochondrial membrane potential was obviously changed in the HG + Pal group, (Fig. 2c) green fluorescence and the red to green ratio was significantly decreased compared with the control group. This change was improved by NaHS treatment (Fig. 2c). To further confirm whether mitochondrial injury caused by HG and palmitate induced endothelial apoptosis, we also tested the expression of cleaved caspase 9 (cl-casp 9), a marker of the mitochondrial apoptosis pathway. The expression of cl-casp 9 was markedly increased after treatment with HG and palmitate. NaHS treatment attenuated this alteration (Fig. 2d). NAC, a scavenger of ROS, had a similar effect on the expression of cl-casp 3, Bax, Bcl-2 and cl-casp 9 (Fig. 2b, d).

### Exogenous H_2_S protected RAECs against autophagy induced by HG and palmitate

**Fig. 3:**
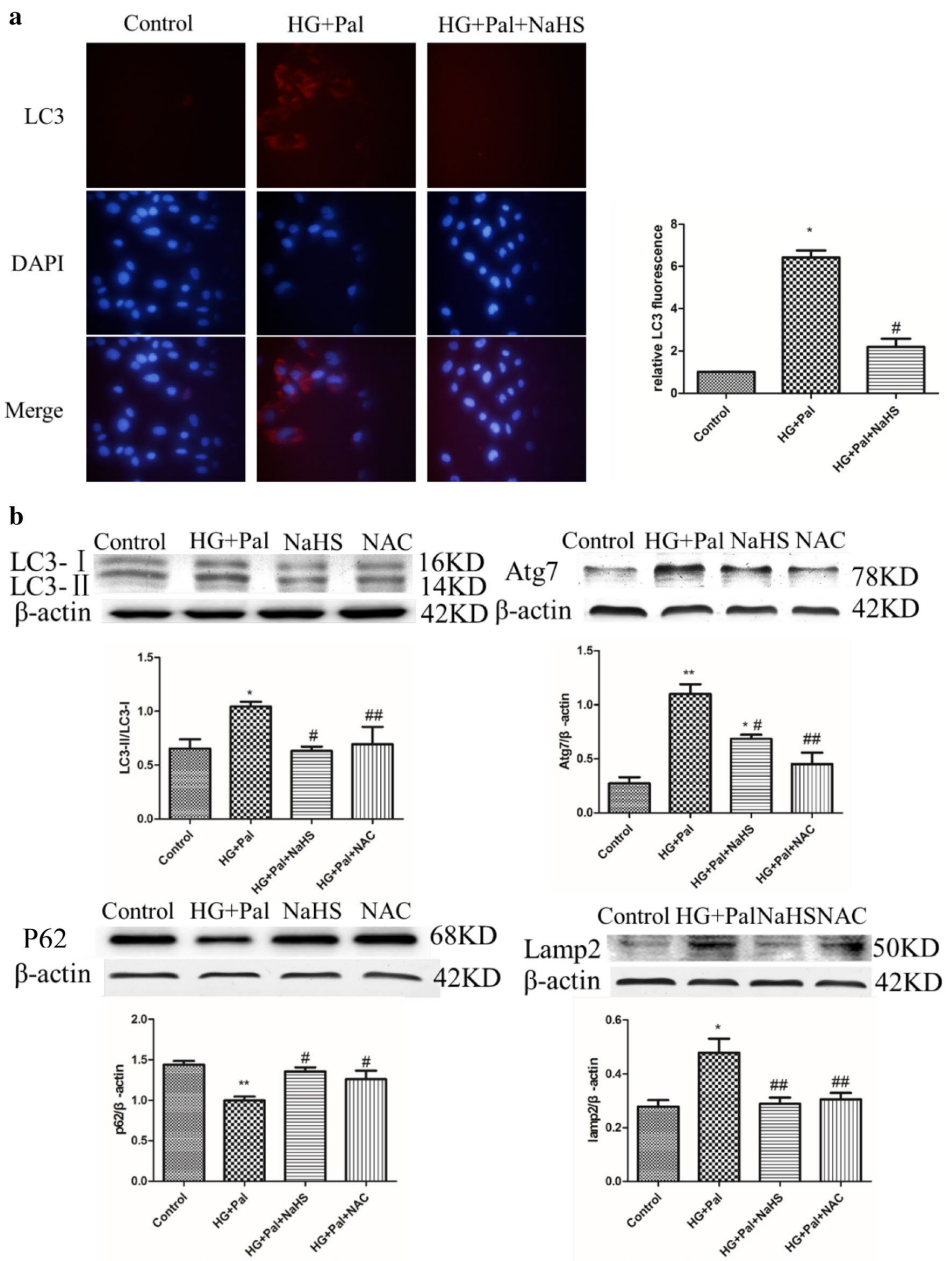
Exogenous H_2_S protected RAECs against apoptosis through attenuating autophagy induced by HG and palmitate. **a** The expression of LC3 was examined by immunofluorescence (*red fluorescence*). The mean fluorescence intensity of *red fluorescence* was measured. There data were summarized from at least three different experiments.(***p < 0.001 vs control group. ^###^p < 0.001 vs HG + Pal group). **b** The expression of LC3II/I, Atg7, P62 and lamp2 was examined by Western blotting. Each OD value was standardized to that of β-actin. There data were summarized from at least three different experiments. (*p < 0.05 vs control group. **p < 0.01 vs control group. ^#^p < 0.05 vs HG + Pal group. ^##^p < 0.01 vs HG + Pal group)

To identify the mechanism through which HG and palmitate treatment could induced arterial endothelial cell apoptosis, we determined the autophagy level. Immunofluorescence was used to test the expression of LC3, an autophagy marker, and it showed that HG and palmitate treatment up-regulated the expression of LC3. This alteration was attenuated by NaHS treatment (Fig. 3a). Though autophagy plays an important role in maintaining cellular homeostasis, excessive autophagy is capable of inducing apoptosis. Our results have demonstrated that HG and palmitate treatment can induce endothelial cell apoptosis. Therefore, we hypothesized that enhanced autophagy levels were closely related to apoptosis in endothelial cells after HG and palmitate treatment. Western blot analysis was also used to examine several other markers of autophagy. It revealed that the markers LC3II/I ratio, Atg7 and lamp2 were up-regulated and that P62 was down-regulated by HG and palmitate (Fig. 3b). To further demonstrate that exogenous H_2_S reverses autophagy, rapamycin, an inhibitor of mTOR, used as autophagy catalyst. Our data confirmed that 100 μM NaHS inhibited expression of LC3II and Atg7 of RAECs treated by rapamycin (Additional file 1: Figure S2). To These data indicated that exogenous H_2_S could protect arterial endothelial cells against apoptosis through suppressing autophagy levels induced by HG and palmitate. NAC also reduced the autophagy level (Fig. 3b).

### Exogenous H_2_S attenuated autophagy by inhibiting activation of AMPK signaling pathway

**Fig. 4:**
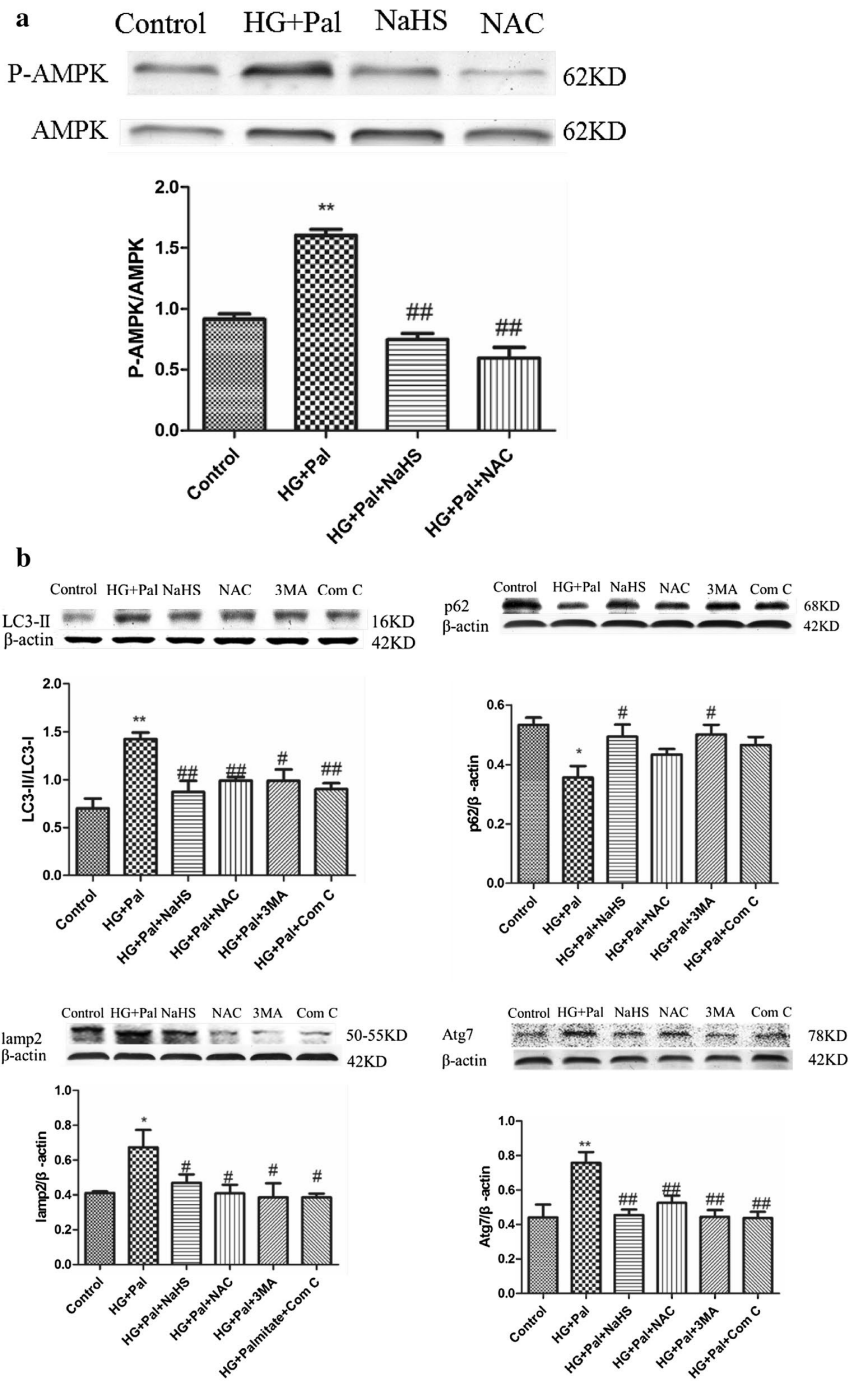
Exogenous H_2_S attenuated autophagy by inhibiting activation of AMPK signaling pathway. **a** The expression of p-AMPK and AMPK was detected by Western blotting. The OD value of P-AMPK was standardized to that of AMPK. There data were summarized from at least three different experiments. (**p < 0.01 vs control group. ^##^p < 0.01 vs HG + Pal group). **b** Compound C inhibited autophagy induced by HG + Pal. The expression of LC3II/I, Atg7, P62 and lamp2 was examined by Western blotting. Each OD value was standardized to that of β-actin. There data were summarized from at least three different experiments (*p < 0.05 vs control group. **p < 0.01 vs control group. ^#^p < 0.05 vs HG + Pal group. ^##^p < 0.01 vs HG + Pal group)

**Fig. 5:**
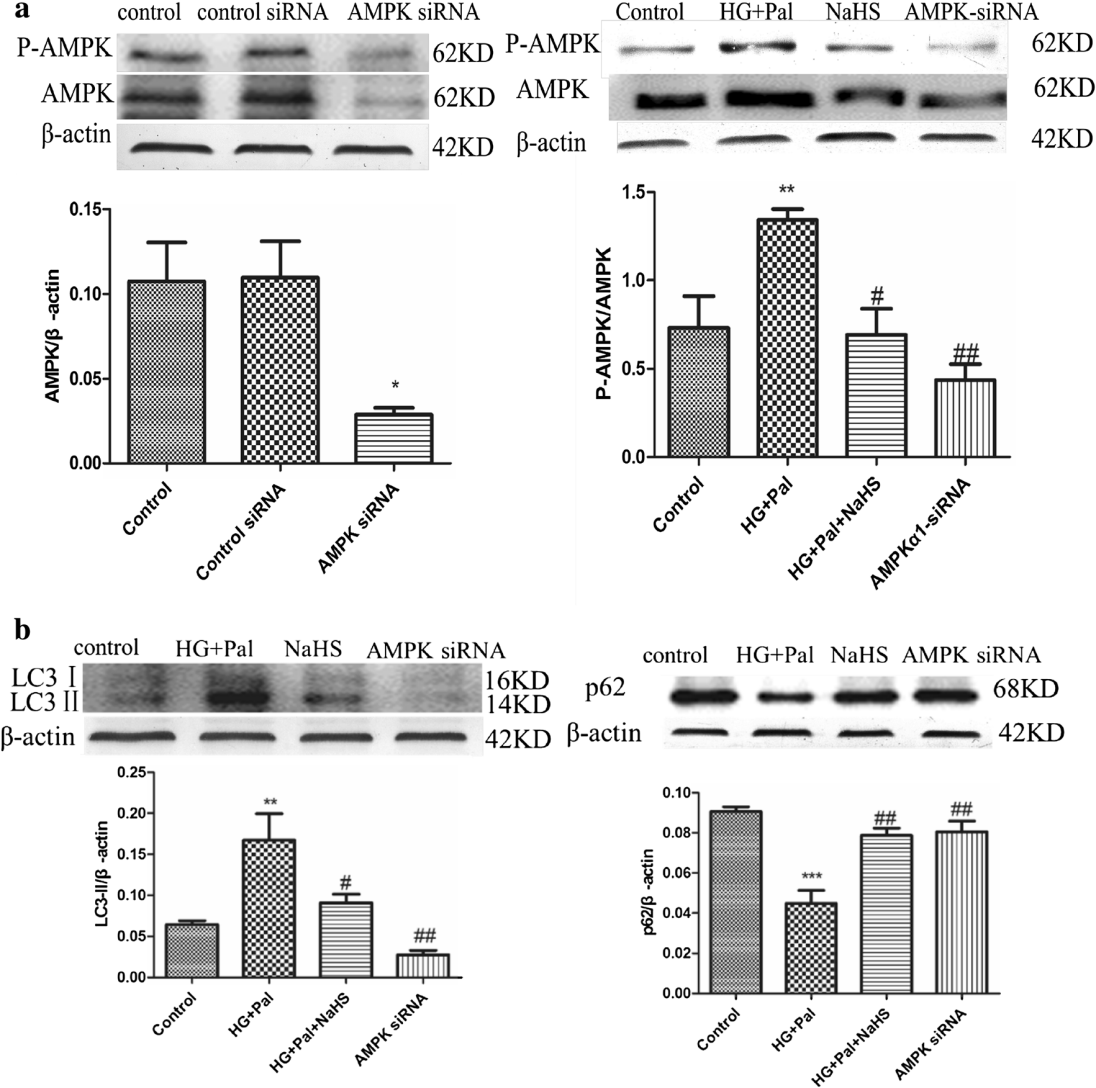
Exogenous H_2_S attenuated autophagy by inhibiting activation of AMPK signaling pathway. **a** AMPK-siRNA inhibited the expression of AMPK. The OD value of AMPK was standardized to that of β-actin. The OD value of P-AMPK was standardized to that of AMPK. There data were summarized from at least three different experiments. (*p < 0.05 vs control group. **p < 0.01 vs control group. ^#^p < 0.05 vs HG + Pal group. ^##^p < 0.01 vs HG + Pal group). **b** AMPK-siRNA attenuated the autophagy level. Each OD value was standardized to that of β-actin. There data were summarized from at least three different experiments. (***p < 0.001 vs control group. ^##^p < 0.01 vs HG + Pal group. and p < 0.05 vs HG + Pal + NaHS group)

Autophagy could be enhanced by the activated AMPK signaling pathway [25]. The p-AMPK to AMPK ratio was detected to confirm the mechanism of the activation of autophagy, and it showed that the HG and palmitate treatment increased the ratio whereas the NaHS and NAC treatment could attenuate this alteration (Fig. 4a). To further investigate whether the AMPK signaling pathway played a role in autophagy induced by HG and palmitate, compound C, an AMPK inhibitor and AMPK-siRNA were applied. Our results showed that compound C had the same effect as NaHS, modifying the expression of LC3, lamp2, P62 and Atg7 (Fig. 4b). AMPK-siRNA significantly inhibited the expression of AMPK (Fig. 5a) and also modified the expression of autophagy associated proteins (Fig. 5b). To reveal that autophagy inhibition can rescue cell death, Atg7 siRNA was treated with RAECs. Western blot analysis showed that the expression of Atg7 was significantly suppressed by Atg7 siRNA compared with that in control siRNA group. The expression of LC3II was reduced in Atg7 silenced RAECs, compared to that in HG and Pal group (Additional file 1: Figure S3). Our results confirmed that knock down of Atg7 could rescue these endothelial cells from oxidative stress induced autophagy.

### Exogenous H_2_S inhibited activation of AMPK through ameliorating mitochondrial injury induce by oxidative stress

**Fig. 6:**
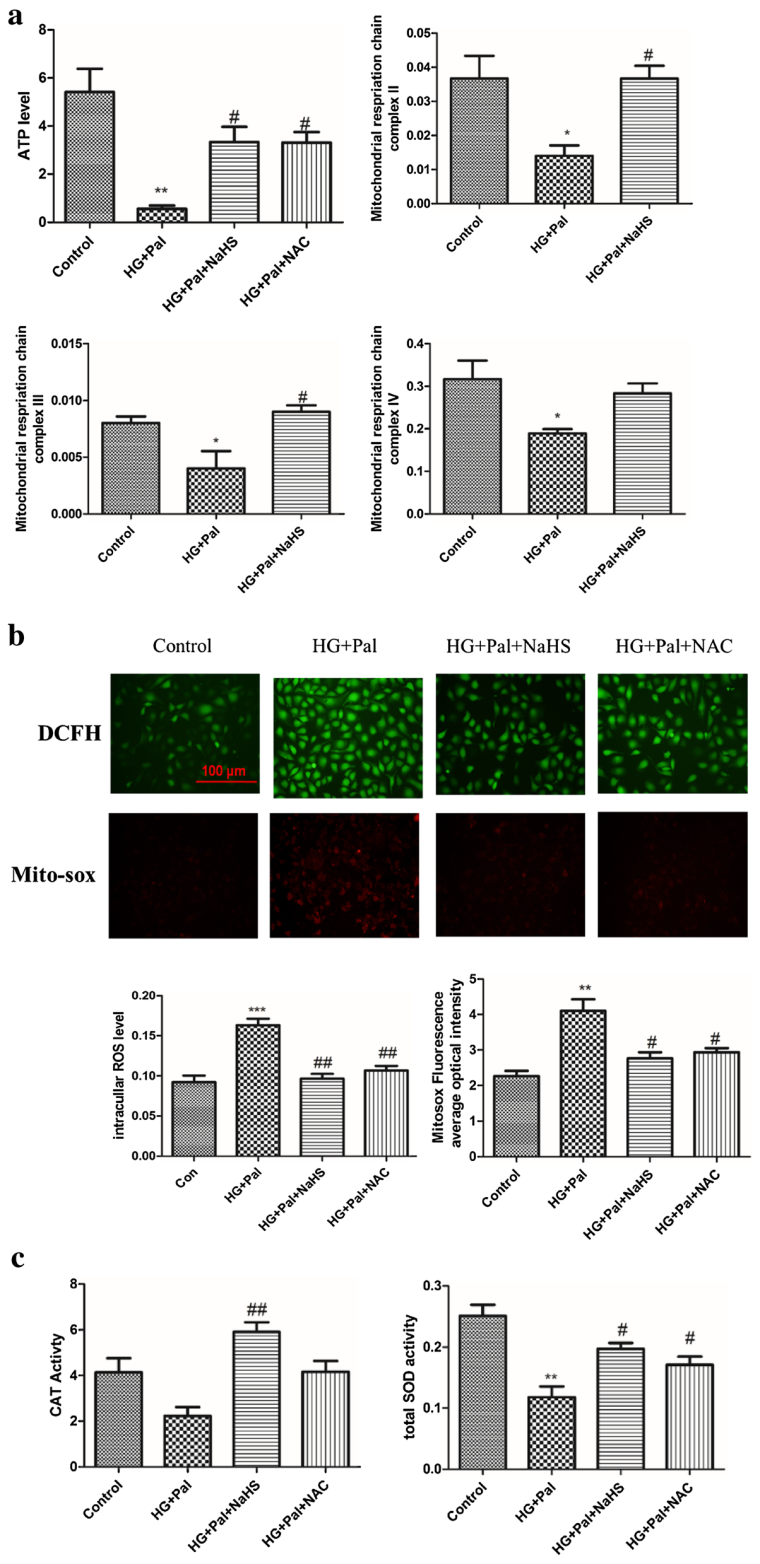
Exogenous H_2_S inhibited activation of AMPK through ameliorating mitochondrial injury induced by oxidative stress. **a** The ATP level and the activities of the mitochondrial respiration chain complex II and III and IV were tested by kits. (*p < 0.05 vs control group. **p < 0.01 vs control group. ^#^p < 0.05 vs HG + Pal group). **b** The whole ROS level was measured by DCFH (*green fluorescence*), and the mitochondrial ROS was measured by mito-SOX (*red fluorescence*). The mean fluorescence intensity was measured. There data were summarized from at least three different experiments. (**p < 0.01 vs control group. ***p < 0.001 vs control group. ^#^p < 0.05 vs HG + Pal group. ^##^p < 0.01 vs HG + Pal group. ^###^p < 0.001 vs HG + Pal group). **c** The activity of CAT and SOD was detected with activity test kits. (*p < 0.05 vs control group. ^#^p < 0.05 vs HG + Pal group. ^##^p < 0.01 vs HG + Pal group)

Reduced ATP production can activate AMPK, which can facilitate autophagy [26]. Hence, we investigated whether exogenous H_2_S was capable of improving mitochondrial function after HG and palmitate treatment. First, we examined ATP production using an ATP assay kit. This result indicated that ATP production was impaired by HG and palmitate and that this adverse effect was improved by NaHS treatment (Fig. 6a). To investigate how exogenous H_2_S plays a role in maintaining ATP production, three enzymes associated with mitochondrial respiratory function, complex II, III and IV were tested. The results suggested that these three enzymes were restrained by HG and palmitate and that NaHS treatment ameliorated these alterations (Fig. 6a).

**Fig. 7:**
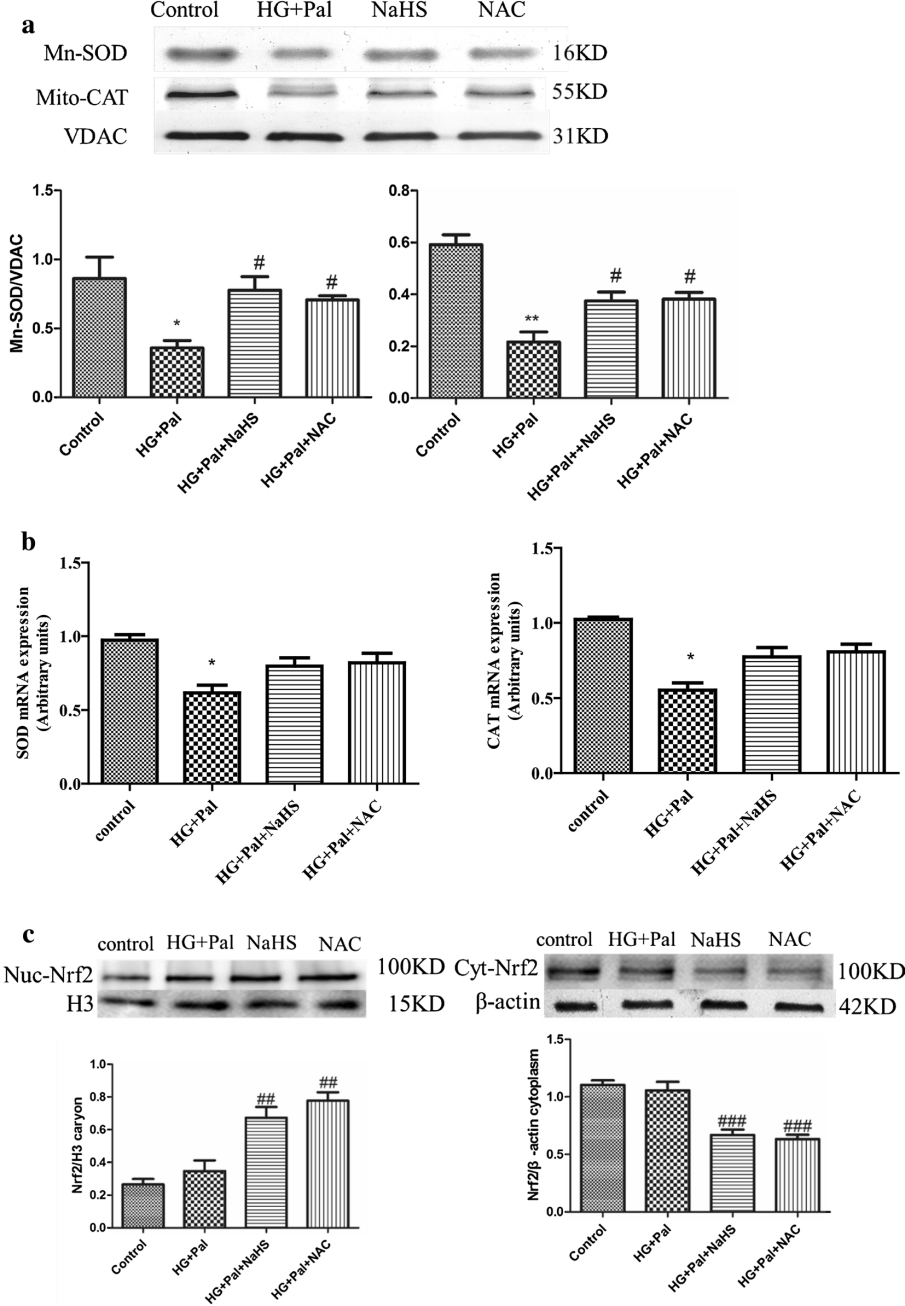
Exogenous H_2_S inhibited activation of AMPK through ameliorating mitochondrial injury induced by oxidative stress. **a** The mitochondrial CAT and SOD level were examined by Western blotting. Each OD value was standardized to that of VDAC. There data were summarized from at least three different experiments. (*p < 0.05 vs control group. **p < 0.01 vs control group. #p < 0.05 vs HG + Pal group). **b** The mRNA levels of SOD and CAT were examined by real-time PCR. (*p < 0.05 vs control group). **c** The expression of Nrf2 in the nucleus and in the cytoplasm was examined by Western blotting. The OD value of nuc-Nrf2 was standardized to that of histone3. The OD value of Cyt-Nrf2 was standardized to that of β-actin. There data were summarized from at least three different experiments. (^##^p < 0.01 vs HG + Pal group. ^###^p < 0.001 vs HG + Pal group)

It has been reported that H_2_S is capable of suppressing ROS production and that ROS scavengers inhibit both apoptosis and autophagy (Figs. 2, 3, 4, 5). Thus, we hypothesized that exogenous H_2_S protected mitochondrial respiratory function through inhibiting ROS production. Therefore, DCFH and mito-sox assays were applied to examine whole ROS and mitochondrial ROS production, respectively. The results showed that both total and mitochondrial ROS production were significantly increased in the HG + Pal group whereas these changes were suppressed by NaHS treatment (Fig. 6b). To test how exogenous H_2_S suppressed ROS production, we tested the activity of CAT and SOD, two ROS scavenger enzymes. Our results showed that the activity of the two enzymes was inhibited by HG and palmitate and rescued by NaHS treatment (Fig. 6c). Because cl-casp 9 and mitochondrial ROS production had a marked change under this scenario (Figs. 2c, 6b), we examined SOD (Mn-SOD) and CAT (mito-CAT) expression in mitochondria. Our data revealed that HG and palmitate impaired Mn-SOD and mito-CAT expression and this alteration was attenuated by NaHS treatment (Fig. 7a). To further test the expression of SOD and CAT, we used real-time PCR to examine the mRNA levels of SOD and CAT. The result of this test indicated that the mRNA levels of SOD and CAT were decreased in the HG + Pal group (Fig. 7b). Mitochondrial dysfunction causes several consequences, including inactivation of mitophagy, ROS level increased and imbalance of calcium. To demonstrate that H_2_S affects mitophagy through AMPK pathway, we detected the expression of parkin and Becline-1. Our data found that the expression of Beclin and Parkin with the treatment of H_2_S and AICAR significantly decreased compared with those of HG and Pal groups (Additional file 1: Figure S4). Our results demonstrated that exogenous H_2_S inhibited mitophagy through AMPK pathway. To demonstrate the effect of NaHS on the relationship between apoptosis and autophagy, the administration of 3-MA and Atg7 siRNA treated with RAECs. Our data showed that the cell death ratio in RAECs treated with NaHS, 3-MA and Atg7 siRNA decreased compared with that in RAECs treated with HG and palmitate group. Our result revealed that apoptosis was possible partly dependent on autophagy (Additional file 1: Figure S5). To further investigate how exogenous H_2_S attenuates this alteration (regulates ROS production) in the scenario, we tested Nrf2 expression, one of the antioxidant signaling pathways. Nrf2 can exert its antioxidant function only when translocated into the nucleus, thus we tested the translocation ratio of Nrf2. The result suggested that the translocation into the nucleus of Nrf2 was not changed when endothelial cells were treated with HG and palmitate, but in contrast, NaHS treatment promoted Nrf2 translocating into the nucleus (Fig. 7c).

## Discussion

The results of the current study provide new insights into the mechanisms of diabetes-induced vascular endothelium injury and reveal an effective protection of exogenous H_2_S in the model. Our results indicate that (i) exogenous H_2_S could attenuate the adhesivity and apoptosis of vascular endothelial cells; (ii) exogenous H_2_S targets suppresses ROS production induced by HG and palmitate and this effect is attributed to promoting Nrf2 translocation into the nucleus, which improves mitochondrial injury, guaranteeing ATP production; (iii) exogenous H_2_S ameliorates excessive activation of autophagy through attenuating the activation of AMPK induced by ATP depletion. Each of these results is discussed in greater detail below.

H_2_S has been proved to exert a wide range of physiological and cytoprotective functions in the biological systems. Among these functions, the role of H_2_S in oxidative stress has been one of the main focuses over years [27]. More and more studies revealed the underlying mechanisms for the antioxidant effect of H_2_S. At 37 °C and pH 7.4, more than 80 % of H_2_S molecules dissolve in surface waters and dissociate into H^+^, HS^−^, and S^2−^ ions. HS^−^ is powerful one-electron chemical reluctant and presents a remarkable capacity to scavenge ROS [28, 29]. Studies have also proven that H_2_S preserves the cellular GSH status and provides protection against oxidative damage in brain [30, 31], spinal cord [32] and heart [33, 34]. Trx-1 was also shown to scavenge ROS and protect cells against oxidative stress. In 2008, Jha et al. reported that H_2_S protected murine liver against ischemia–reperfusion (I/R) injury through upregulation of intracellular Trx-1 along with an increase in hepatic tissue GSH/GSSG ratio [35]. Therefore, H_2_S can stimulate cellular enzymatic or nonenzymatic antioxidants to scavenge free radicals.

Diabetes-induced vascular injury is a major complication, which is induced by either hyperglycemia or hyperglycemia coupled with hyperlipidemia [36, 37]. H_2_S plays an important role in diabetes and its complications [38]. Our previous study has shown that CSE, a H_2_S generated enzyme, is down-regulated in diabetic rat cardiac tissue [39]. In the current study, we used db/db mice, a type II diabetes model, to detect the generation of endogenous H_2_S in arteries. We found that the generation of H_2_S and the expression of CSE were reduced in type II diabetes arteries (Fig. 1a, b). Endothelial cells play a pivotal role in mediating the function of arteries. So we specially examined the effect that HG and palmitate treatment had on the H_2_S generation of arterial endothelial cells. These results told us that the H_2_S generation of arterial endothelial cells was impaired by HG and palmitate (Fig. 1c, d). Therefore, we investigated whether an exogenous H_2_S treatment could cause resistance to arterial endothelial injury in type II diabetes. Three endothelial dysfunction markers, vWF, ITGβ1 and GP1βA were down-regulated by NaHS treatment compared with type II diabetes both in vivo and in vitro (Fig. 1e). NaHS treatment also reversed the trend in the expression of CSE (Fig. 1b, d). These results indicate that exogenous H_2_S is capable of protecting the function of arterial endothelial cells.

The arterial endothelial cell apoptosis is the main reason of diabetes-induced vascular dysfunction [40, 41]. The TUNEL and Hoechst/PI assays were utilized to examine apoptosis of arterial endothelial cells. We observed that the number of apoptotic cells was significantly increased by the HG and palmitate treatment and that the NaHS treatment reduced the number of apoptotic cells (Fig. 2a). The activation of caspase 3 was suppressed by the NaHS treatment (Fig. 2b cl-casp3). Bax and Bcl-2 belong to the Bcl family and are two main apoptosis mediating factors that act through the Bcl-2/Bax signaling pathway [42–44]. Exogenous H_2_S was capable of up-regulating Bcl-2 and down-regulating Bax, which inhibited apoptosis (Fig. 2b). To induce apoptosis, Bax is separated from Bcl-2 and then accumulates on mitochondria which changes mitochondrial membrane potential [45] resulting in the release of cytochrome C and mitochondrial DNA, which activates the mitochondrial apoptosis pathway [46]. Therefore, the JC-1 assay was used to detect mitochondrial membrane potential. We found that the mitochondrial membrane potential was significantly decreased in cells under HG and HG with palmitate compared with the control group (Fig. 2c). Bax expression increased, Bcl-2 expression decreased and the changed mitochondrial membrane potential changed which caused up-regulation of the expression of cleaved caspase 9, which activated mitochondrial apoptosis pathway (Fig. 2d). NaHS attenuated the change in mitochondrial membrane potential and the expression of cl-casp 9 (Fig. 2d), which indicated that exogenous H_2_S was capable of maintaining mitochondrial function and preventing mitochondrial apoptosis in arterial endothelial cells.

Previous studies have shown that oxidative stress plays a pivotal role in diabetic vascular injury [36], and the excessive ROS production is a cause of mitochondrial injury [47, 48]. H_2_S can suppress ROS production as a ROS scavenger [49] and increase SOD activity in cardiomyocytes [50]. So we focused on whether exogenous H_2_S could suppress ROS production in arterial endothelial cells. Our results indicated that NaHS suppressed both whole ROS and mitochondrial ROS production (Fig. 6b). The effect of exogenous H_2_S on ROS production was partly attributed to an increase in the activity of SOD and catalase (CAT) (Fig. 6c). Furthermore, exogenous H_2_S markedly increased mitochondrial Mn-SOD and mito-CATexpression (Fig. 7a), which showed that exogenous H_2_S could suppress mitochondrial ROS production a step further. We also found that NAC, a ROS scavenger, had a similar effect on SOD and CAT (Figs. 6c, 7a), which suggested that the suppression of ROS and the activity of SOD and CAT promoted each other. Furthermore, HG and palmitate treatment resulting in excessive oxidative stress could induce the disruption of mitochondrial respiratory function and ATP depletion. NaHS could ameliorate the dysfunction of mitochondria (Fig. 6a).

ATP depletion within cells can active the AMPK signaling pathway [26], which interacts with ULK1 to induce autophagy [25]. Autophagy is an important catabolic program in the maintenance of cellular homeostasis; it handles a variety of macromolecular cellular contents, ranging from protein aggregates, dysfunctional subcellular organelles, infected pathogens, and the storage of nutrients [51–53]. However, continuous activation of AMPK results in excessive autophagy, which plays an important role in apoptosis [54, 55]. We found that the AMPK pathway was activated by HG and palmitate treatment (Fig. 4a) and, in response to the activation, the autophagy of endothelial cells was significantly increased (Fig. 3a, b). Though NaHS treatment restrained autophagy on the whole, it could not return it to normal levels compared with the control group (Fig. 3a). The expression of Atg7 was significantly higher than in controls (Fig. 3b), which indicated that exogenous H_2_S could mediate autophagy on a certain level to maintain cellular homeostasis. NAC treatment had a similar effect on autophagy to NaHS, which suggested that ROS played a crucial role in the activation of excessive autophagy (Fig. 3b). As an activator of autophagy, AMPK was also inhibited by NaHS treatment (Fig. 4a). To investigate whether AMPK has a strong effect on autophagy, AMPK siRNA was applied. The results indicated that AMPK siRNA suppressed the expression and activation of AMPK, which restrained activation of autophagy (Fig. 5a, b). The inhibition of autophagy by AMPK siRNA occurred to a greater extent than the inhibition caused by NaHS (Fig. 5b), which again indicated that exogenous H_2_S could mediate autophagy on a certain level. Autophagy and apoptosis control the turnover of organelles and proteins within cells, and of cells within organisms, respectively, and many stress pathways sequentially elicit autophagy, and apoptosis within the same cell. Generally autophagy blocks the induction of apoptosis, and apoptosis-associated caspase activation shuts off the autophagic process. However, in some cases, autophagy or autophagy-relevant proteins may help to induce apoptosis or necrosis. There are also several examples in which the induction of autophagy facilitates the activation of apoptosis [56].

Nrf2 is a key transcriptional regulatory protein that activates antioxidant enzyme genes [57]. In the cytoplasm, Nrf2 couples with Kelch-like ECH associating protein 1 (Keap-1), which inhibits its transfer into the nucleus. H_2_S creates the process of s-sulfhydrate Keap1 releasing Nrf2, which promotes Nrf2 translocation into the nucleus [58]. We found that the transfer of Nrf2 was not changed when endothelial cells were treated with HG and palmitate and that NaHS treatment promoted Nrf2 nuclear transfer in accordance with previous studies (Fig. 7c).

## Conclusions

**Fig. 8:**
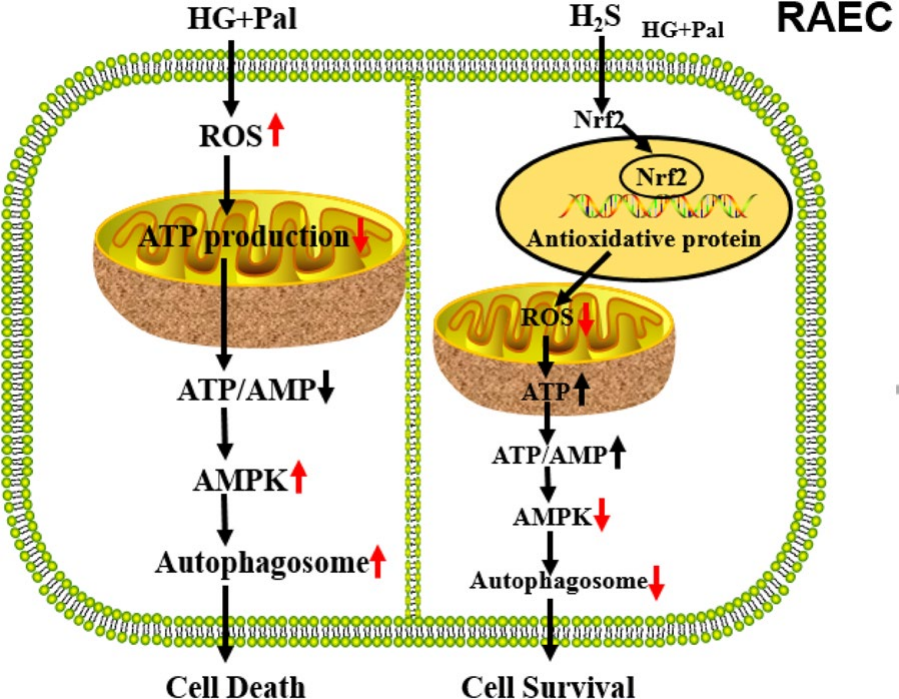
The protective effect of H_2_S on vascular endothelial cells in type II diabetes model. HG and palmitate induces an increase in ROS production in vascular endothelial cells. Increased ROS leads to extremes oxidative stress which impairs ATP production. This alteration results in excessive autophagy, which finally induces cell death. Exogenous H_2_S attenuates ROS production through the Nrf2-ROS-AMPK pathway. Through attenuating ROS production, H_2_S inhibits this pathway, which reduces excessive autophagy. This effect of H_2_S can maintain the homeostasis of vascular endothelial cells and increase cell survival

In summary, our results demonstrated that exogenous H_2_S could suppress oxidative stress induced by HG and palmitate in arterial endothelial cells through promoting Nrf2 nuclear transfer. This exerts a protective effect against mitochondrial injury, which maintains ATP production. High glucose/palmitate-induced arterial endothelial cells injury due to oxidative stress induced mitochondrial injury, at least in part. Mitochondrial injury results in ATP depletion, which activates the AMPK signaling pathway. Continuous and excessive activation of the AMPK signaling pathway leads to excessive autophagy, which possibly results in arterial endothelial apoptosis. Exogenous H_2_S not only rescues the endogenous of H_2_S but also protects arterial endothelial cells against cell death (Fig. 8). Upon description of the mechanism conferred by exogenous H_2_S protection, it is possible to provide a new avenue for therapeutic opportunities for diabetes-induced arterial endothelial injury.

## Additional file

10.1186/s13578-016-0099-1 NaHS effected on autophagy and apoptosis of RAECs with the treatment of HG and Pal. **Figure S2.** RAECs were treated with HG and Pal in the presence of autophagy activator (rapamycin) to determine the expression of LC3II/I and Atg7 by western blot analysis. **Figure S3.** Atg7 siRNA reducing the expression of LC I/II in RAECs treated with HG and Pal. **Figure S4.** RAECs were treated with HG and Pal and NAHS in presence of AMPK catalyst (AICAR) to determine the expression of mitophage associated proteins (Parkin and Beclin 1) by western blot analysis. **Figure S5.** The cell death of RAECs treated by HG and Pal with 3-MA and Atg7 siRNA was measured by Hoechst 33342/PI staining.
