# Supplementary material for: Hydrogen sulfide decreases high glucose/palmitate-induced autophagy in endothelial cells by the Nrf2-ROS-AMPK signaling pathway
Source: Cell Biosci. 2016 May 23;6:33. doi: 10.1186/s13578-016-0099-1 (PMC4877995; doi:10.1186/s13578-016-0099-1)
Supplement: Supplementary file 1 — 10.1186/s13578-016-0099-1 NaHS effected on autophagy and apoptosis of RAECs with the treatment of HG and Pal. Figure S2. RAECs were treated with HG and Pal in the presence of autophagy activator (rapamycin) to determine the expression of LC3II/I and Atg7 by western blot analysis. Figure S3. Atg7 siRNA reducing the expression of LC I/II in RAECs treated with HG and Pal. Figure S4. RAECs were treated with HG and Pal and NAHS in presence of AMPK catalyst (AICAR) to determine the expression of mitophage associated proteins (Parkin and Beclin 1) by western blot analysis. Figure S5. The cell death of RAECs treated by HG and Pal with 3-MA and Atg7 siRNA was measured by Hoechst 33342/PI staining. [file 13578_2016_99_MOESM1_ESM.pdf]

Supplement

Methods

Cell culture and treatment

Primary cultures of rat aortic endothelial cells (RAECs), purchased from Chinese Academy of Sciences Cell Bank, were grown as monolayers at a density of 5×10^4 cells/cm^2 in Dulbecco's modified Eagle medium (DMEM) and incubated at 37℃ in humidified air containing 5% CO_2. The medium contained 10% calf serum, 100 units/ml penicillin and 100 μg/ml streptomycin. Two days after seeding, the cultured RAEC were randomly divided into the following six groups and treatments: control group (low glucose, LG, 5.5 mmol/L), high glucose (HG, 40 mM) + Palmitate (Pal, 500μM), HG+Pal+NaHS (100 μM), HG+Pal+NAC (10 mM, an inhibitor of reactive oxygen species), HG+Pal+NaHS+rapamycin (5mM, as an autophagy catalyst) andHG+Pal+NaHS+AICAR (5-aminoimidazole-4-carboxamide ribonucleoside, 500μM, an catalyst of AMPK). Drugs were added directly to the culture for 48 h.

Atg 7 siRNA transfection

The RAECs (80% confluent) were treated according to the manufacturer's instructions with Atg 7 short interfering RNAs (siRNAs) (mouse; Santa Cruz Biotechnology) for 48 h to inhibit Atg 7 expression. Transfected cells were confirmed by Western blot analysis of Atg 7 protein expression.

Statistical analysis

Data are expressed as the mean ± standard error (SEM). Statistical analysis was analyzed by one-way ANOVA. Differences between individual groups were analyzed using Student's t-test. $P < 0.05$ was considered statistically significant and $p < 0.01$ was considered very significant.

Results

1. NaHS effecting on autophagy and apoptosis of RAECs with the treatment of HG and Pal by dose- and time-dependant ways.

To demonstrate that NaHS, as an exogenous H_2S donor, effects on autophagy and apoptosis in rat aortic endothelial cells(RAECs) in different time points and different doses, with the treatment of high glucose and palmitate, MDC and Hoechst33342/PI assays were used in our experiments .Our results revealed that 100μM and 200μM NaHS significantly decreased autophagy (Figure.S1B), apoptotic rates and necrotic rates (Figure.S1D) in RAECs with the treatment of HG and Pal compared with those in 50μM and 500μM NaHS groups. We also observed that NaHS affected autophagy and apoptotic and necrotic rates in RAECs with the treatment of HG and Pal in time-dependent manner, our data showed that 100μM NaHS inhibited autophagy (Figure.S1A), apoptotic and necrotic rates (Figure.S1C) in RAECs for 48h and 72h compared with those in HG and Pal group for 24h. Therefore, we chose 100μM NaHS to apply for 48h in our experiments.

2. Exogenous H_2S regulating autophagy in RAECs treated with HG and Pal

Rapamycin was used, an inhibitor of mTOR, as an autophagy catalyst to determine if rapamycin could reverse autophagy of RAECs with the H_2S treatment, the expression of LC3 I/II and Atg7 was analyzed by Western blot. Our results demonstrated that pretreatment with 100μM NaHS of RAECs inhibited the expression of LC3II and Atg7 of RAECs with the treatment of Rapamycin. Exogenous H_2S could reduce autophagy of RAECs with the treatment of HG and palmitate and rapamycin(Figure.S1).

3. Inhibition of autophagy in RAECs with the treatment of HG and Pal by Atg 7 siRNA

Atg7, an E1-like enzyme, is essential for the autophagy conjugation system, the formation of autophagosomes and starvation-induced degradation of proteins and organelles in mammalian cells [6,7]. Atg7 was knocked down by siRNA silencing in

RAECs, Western blot analysis showed that the expression of Atg7 was significantly suppressed by Atg7 siRNA compared with that in control siRNA group. To validate Atg7 being involved in autophagy induced by exogenous H_2S, the expression of LC3II was reduced in Atg7 silenced RAECs, compared to that in HG and Pal group (Figure.S3). Our results confirmed that knock down of Atg7 could rescue these endothelial cells from oxidative stress induced autophagy.

4. Exogenous H_2S affects mitophagy through AMPK pathway

Parkin can play a critical role in mitophagy in mammalian cells. Some studies found that the intracellular location of Parkin is regulated by mitochondrial function. Parkin normally residues in the cytosol but it translocates to depolarized mitochondria[1]. Mitochondrial localized Parkin promoting the colocalization of mitochondria is observed in cells, which increases oxidative stress[2]. Beclin 1,the first identified mammalian autophagy protein [3], can function as a tumor suppressor in mammals and interacts with the anti-apoptotic Bcl-2 family proteins Bcl-2 and Bcl-XL but not Bax [4,5]. We investigated mitophagy-associated protein expression. The expression of Beclin and Parkin was analyzed by western blot. Our data found that the expression of Beclin and Parkin with the treatment of H_2S and AICAR significantly decreased compared with those of HG and Pal groups (Figure.S4). Our results demonstrated that exogenous H_2S inhibited mitophagy through AMPK pathway.

5. **The effect of NaHS on the relationship between apoptosis and autophagy**

The administration of 3-MA, an autophagy inhibitor, treated with RAECs. Our data showed that the cell death ratio(Figure.S5A) and the expression of cl-caspase-3 (Figure.S5B) in RAECs treated with NaHS, NAC and 3-MA decreased compared with that in RAECs treated with HG and palmitate group. To further demonstrate that autophagy inhibitor can rescue cells from HG+Pal-induced death. The cell death of RAECs treated by HG and Pal with Atg 7 siRNA was measured by Hoechst33342/PI staining. Our result showed that cell death ratio was higher in HG and Pal group that

in Atg 7 siRNA, NaHS and NAC groups (Figure.S5C). Our result revealed that apoptosis was possible partly dependent on autophagy.

References：

1. Y. Chen, G.W. Dorn 2nd., PINK1-phosphorylated mitofusin 2 is a Parkin receptor for culling damaged mitochondria, Science 340 (2013) 471–475.

2. M. Iguchi, Y. Kujuro, K. Okatsu, F. Koyano, H. Kosako, M. Kimura, et al., Parkin Catalyzed ubiquitin-ester transfer is triggered by PINK1-dependent phosphorylation, J.Biol. Chem. 288(2013) 22019–22032.

3. Liang, X. H., Jackson, S., Seaman, M., Brown, K., Kempkes, B., Hibshoosh, H., and Levine, B. (1999) Nature 402, 672–676

4. Aita, V. M., Liang, X. H., Murty, V. V., Pincus, D. L., Yu, W., Cayanis, E., Kalachikov, S., Gilliam, T. C., and Levine, B. (1999) Genomics 59, 59–65

5. Qu, X., Yu, J., Bhagat, G., Furuya, N., Hibshoosh, H., Troxel, A., Rosen, J., Eskelinen, E. L., Mizushima, N., Ohsumi, Y., Cattoretti, G., and Levine, B. (2003) J. Clin. Investig. 112, 1809–1820

6. Klionsky DJ. 2007. Autophagy: from phenomenology to molecular understanding in less than a decade. Nat. Rev. Mol. Cell Biol. 8:931–937.

7. Komatsu M, Wang QJ, Holstein GR, Friedrich VL, Jr, Iwata J, Kominami, E, Chait BT, Tanaka K, Yue Z. 2007. Essential role for autophagy protein Atg7 in the maintenance of axonal homeostasis and the prevention of axonal degeneration. Proc. Natl. Acad. Sci. U. S. A. 104:14489– 14494.

Figure legends

Figure.S1 NaHS effected on autophagy and apoptosis of RAECs with the treatment of HG and Pal. (A) RAECs were treated with high glucose and palmitate, HG and Pal plus 100μM NaHS, and HG and Pal plus NAC for 24h, 48h, 72h, representative images showed bright dotted structure (autophagosomes) that accumulated in cells stained with MDC. Original magnification:40x. (B) RAECs were treated by HG and Pal with 50μM, 100μM, 200μM, and 500μM NaHS, respectively. (C and D) Representative photographs of double staining of PI and Hoechst 33342. The apoptotic cells were observed as nuclei pyknosis by Hoechst 33342. PI –positive cells (red) are regarded as the necrotic cells. RAECs were treated with high glucose and palmitate, HG and Pal plus 100μM NaHS, in time dependent manner(C) and in dose-dependent manner (D). The data represent the mean value ± SE of independent experiments,*p<0.05 vs control.**p<0.01 vs control, #p<0,05 vs HG and Pal

Figure.S2 RAECs were treated with HG and Pal in the presence of autophagy activator (rapamycin) to determine the expression of LC3II/I and Atg7 by western blot analysis. The data represent the mean value ± SE of independent experiments,*p<0.05 vs HG and Pal group.

Figure.S3 Atg 7 siRNA reducing the expression of LC I/II in RAECs treated with HG and Pal..(A) Atg7-siRNA inhibited the expression of Atg7.(B) Atg7 siRNA reduced the expression of LC3I/II in RAECs with the treatment of HG and Pal. *p<0.05 vs control group, ***p<0.001 vs control siRNA group.

Figure.S4. RAECs were treated with HG and Pal and NaHS in presence of AMPK catalyst (AICAR) to determine the expression of mitophage associated proteins (Parkin and Beclin 1) by western blot analysis. *p<0.05 vs Control group.

Figure.S5 The cell death of RAECs treated by HG and Pal with 3-MA and Atg 7 siRNA was measured by Hoechst33342/PI staining. (A) RAECs were treated by HG and Pal with3-MA. Representative photographs of double staining of PI and Hoechst 33342. The apoptotic cells were observed as nuclei pyknosis by Hoechst 33342. PI –positive cells (red) are regarded as the necrotic cells. (B) The expression of cl-caspase-3 in RAECs with the treatment of HG and palmitate. (C) The cell death of RAECs was treated by HG and Pal with Atg 7 siRNA transfection. The results were expressed as the mean ±SD. (* p< 0.05 vs control group, n=4)

Figure.S1

A

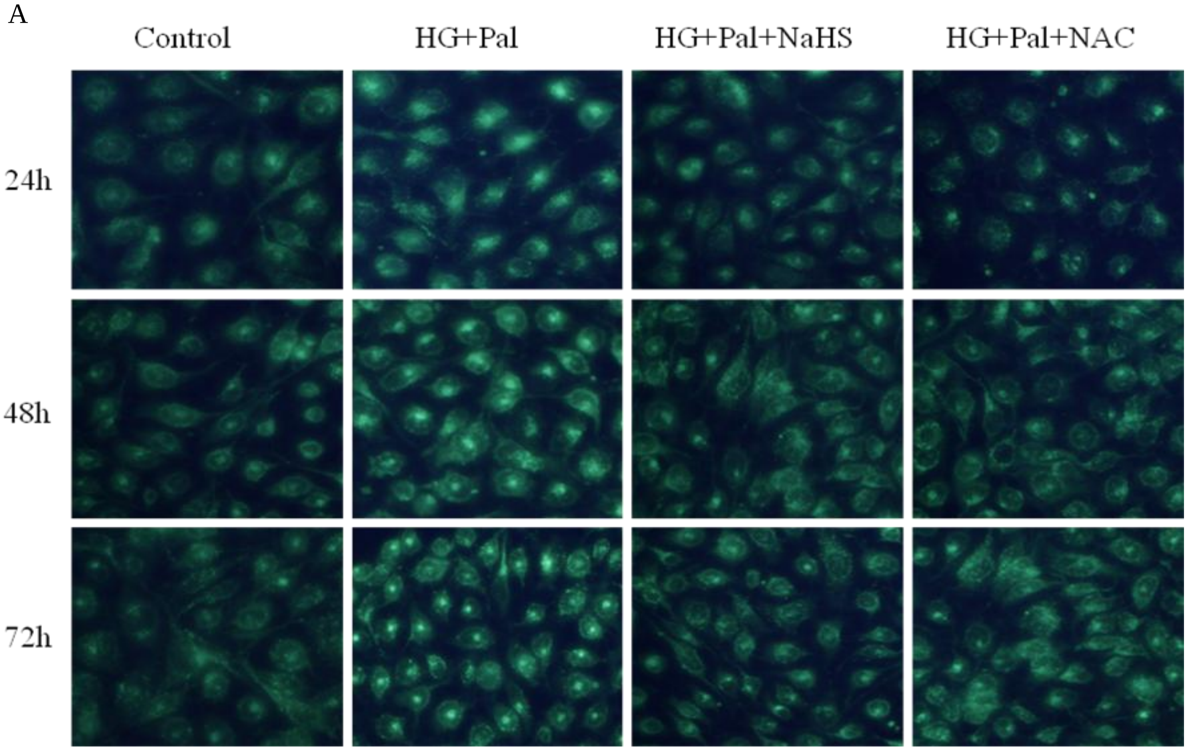
Control
HG+Pal
HG+Pal+NaHS
HG+Pal+NAC
24h
48h
72h

B

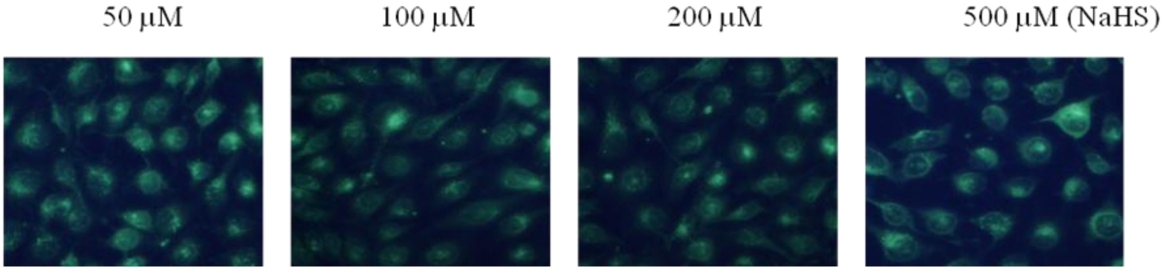
50 μM
100 μM
200 μM
500 μM (NaHS)

C

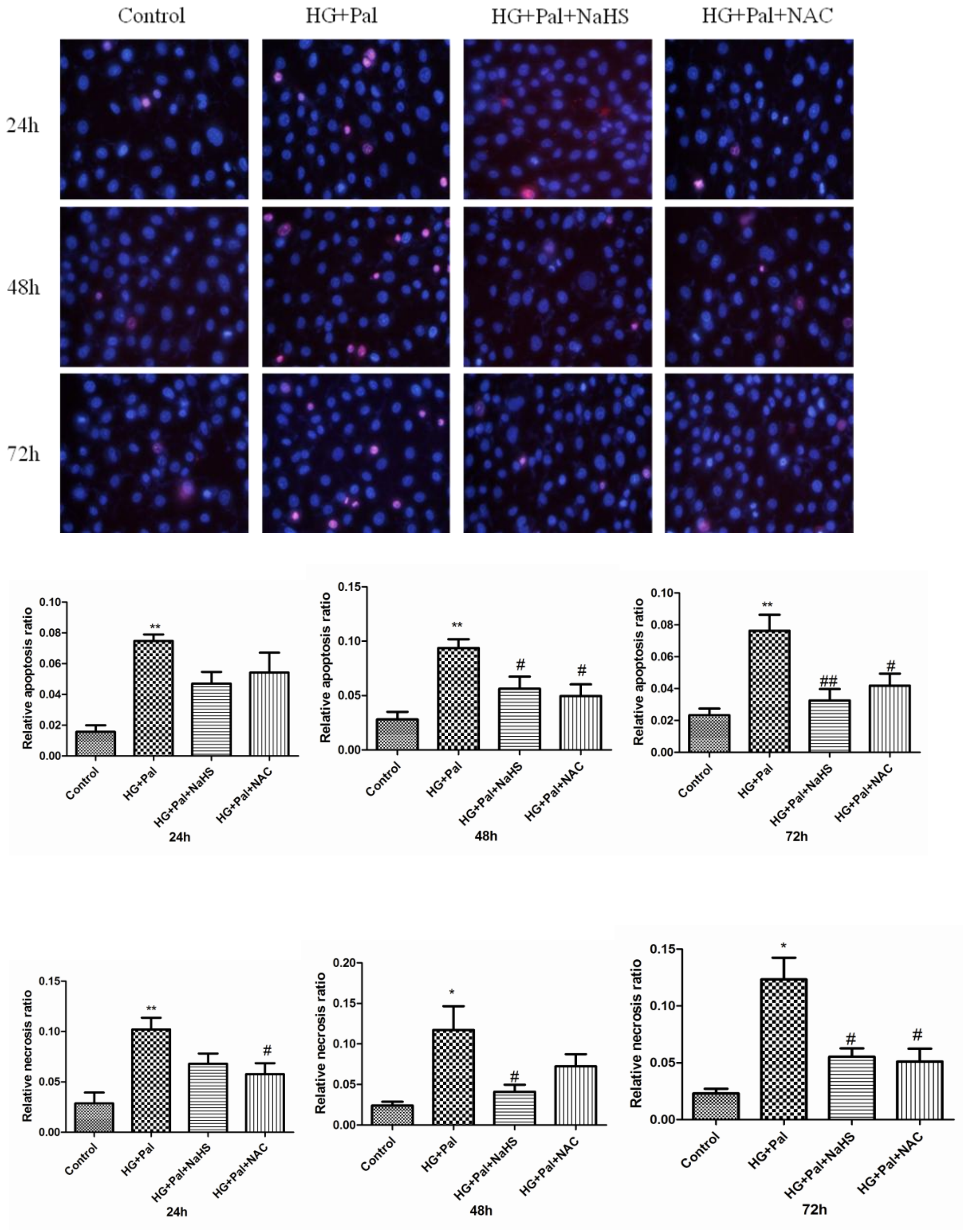

Control
HG+Pal
HG+Pal+NaHS
HG+Pal+NAC
24h
48h
72h
Relative apoptosis ratio
0.00
0.02
0.04
0.06
0.08
0.10
**
Control
HG+Pal
HG+Pal+NaHS
HG+Pal+NAC
24h
Relative apoptosis ratio
0.00
0.05
0.10
0.15
**
#
#
Control
HG+Pal
HG+Pal+NaHS
HG+Pal+NAC
48h
Relative apoptosis ratio
0.00
0.02
0.04
0.06
0.08
0.10
**
##
#
Control
HG+Pal
HG+Pal+NaHS
HG+Pal+NAC
72h
Relative necrosis ratio
0.00
0.05
0.10
0.15
**
#
Control
HG+Pal
HG+Pal+NaHS
HG+Pal+NAC
24h
Relative necrosis ratio
0.00
0.05
0.10
0.15
0.20
*
#
Control
HG+Pal
HG+Pal+NaHS
HG+Pal+NAC
48h
Relative necrosis ratio
0.00
0.05
0.10
0.15
*
#
#
Control
HG+Pal
HG+Pal+NaHS
HG+Pal+NAC
72h

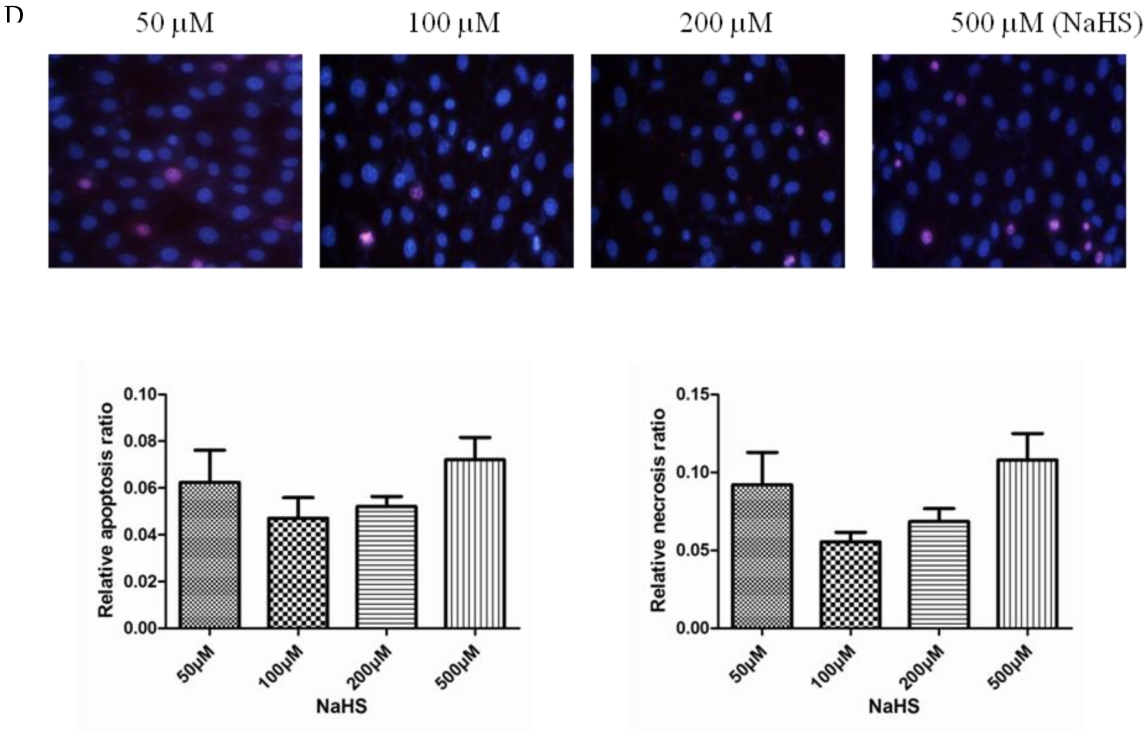
D
50 μM
100 μM
200 μM
500 μM (NaHS)
Relative apoptosis ratio
0.10
0.08
0.06
0.04
0.02
0.00
50μM
100μM
200μM
500μM
NaHS
Relative necrosis ratio
0.15
0.10
0.05
0.00
50μM
100μM
200μM
500μM
NaHS

Figure.S2

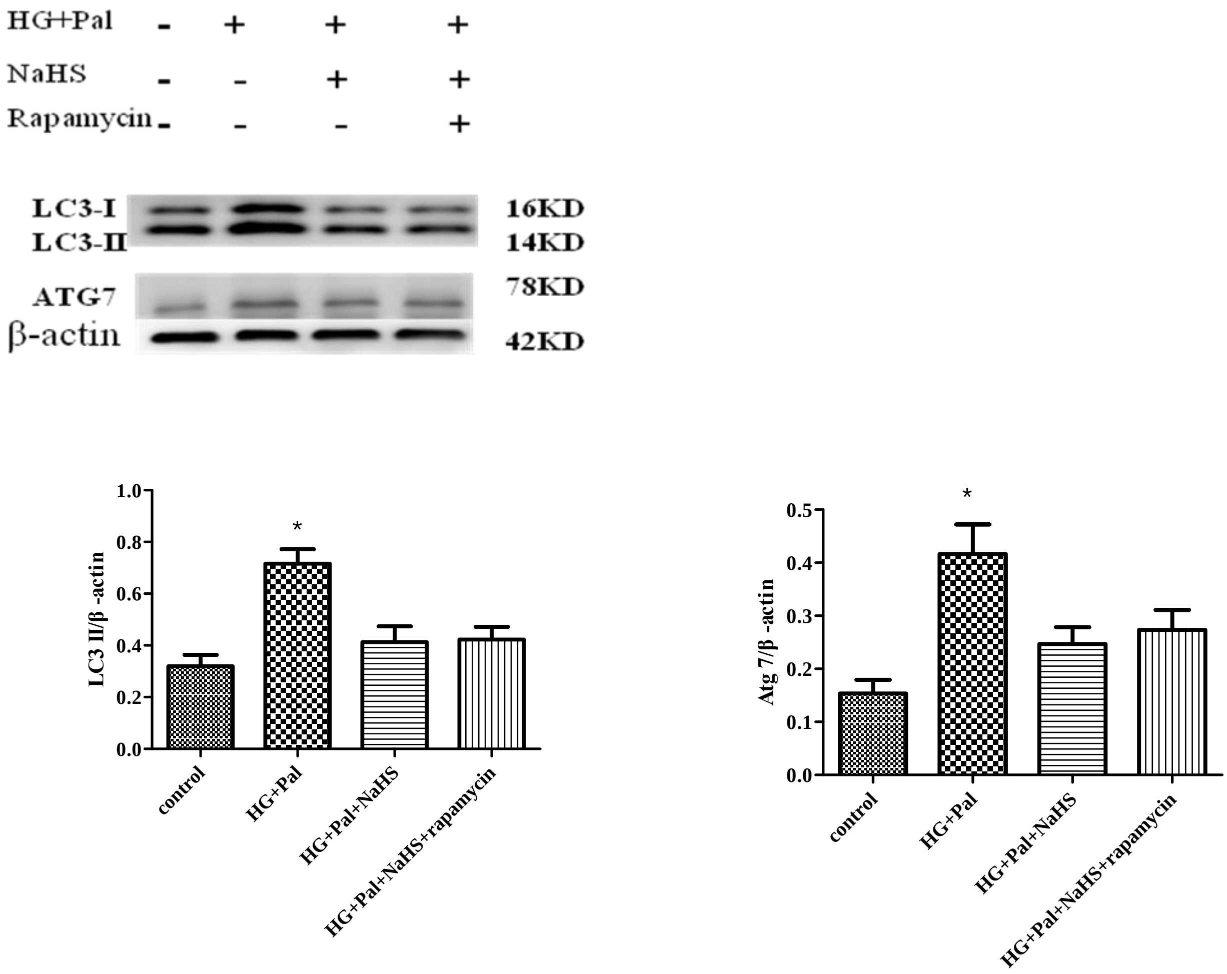
HG+Pal - + + +
NaHS - - + +
Rapamycin - - - +
LC3-I
LC3-II
16KD
14KD
ATG7
78KD
β-actin
42KD
LC3 II/β -actin
1.0
0.8
0.6
0.4
0.2
0.0
*
control
HG+Pal
HG+Pal+NaHS
HG+Pal+NaHS+rapamycin
Atg 7/β -actin
0.5
0.4
0.3
0.2
0.1
0.0
*
control
HG+Pal
HG+Pal+NaHS
HG+Pal+NaHS+rapamycin

Figure.S3

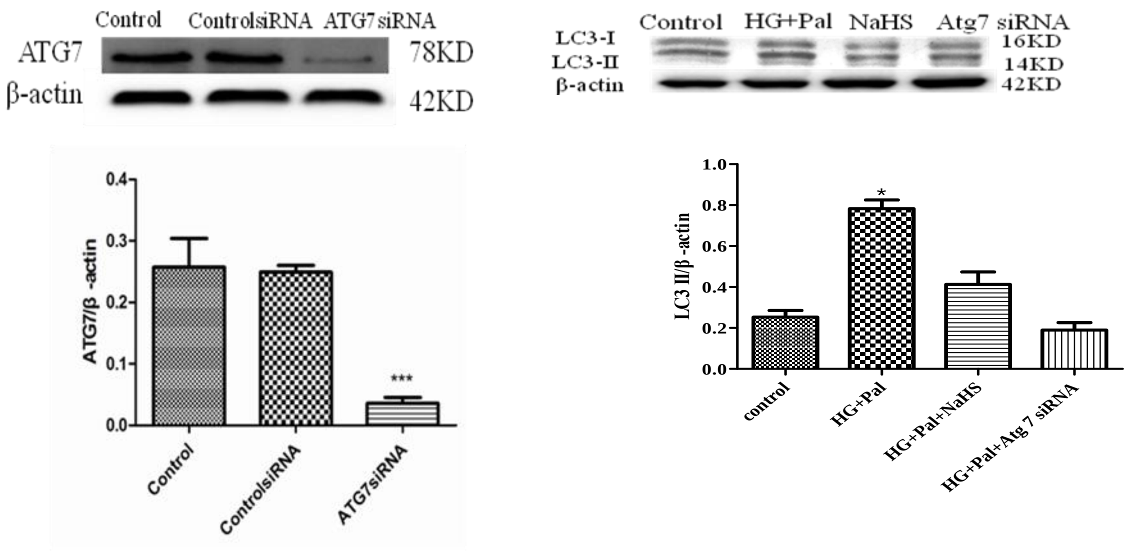
Control
Controlsi RNA
ATG7 siRNA
ATG7
78KD
β-actin
42KD
ATG7/β -actin
0.4
0.3
0.2
0.1
0.0
***
Control
ControlsiRNA
ATG7siRNA
Control
HG+Pal
NaHS
Atg7 siRNA
LC3-I
LC3-II
β-actin
16KD
14KD
42KD
LC3 II/β -actin
1.0
0.8
0.6
0.4
0.2
0.0
*
control
HG+Pal
HG+Pal+NaHS
HG+Pal+Atg 7 siRNA

Figure.S4

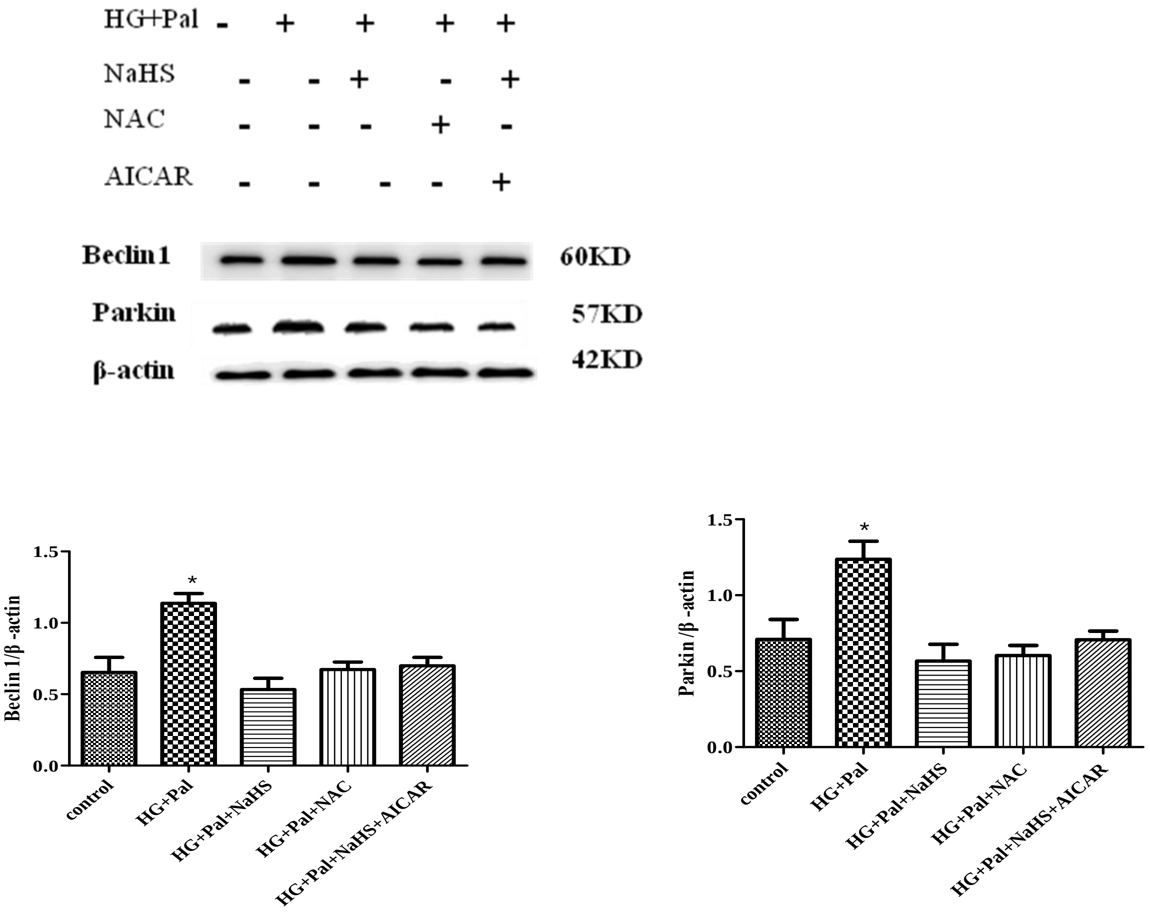

HG+Pal - + + + +
NaHS - - + - +
NAC - - - + -
AICAR - - - - +
Beclin1
60KD
Parkin
57KD
β-actin
42KD
Beclin 1/β -actin
Parkin /β -actin
control
HG+Pal
HG+Pal+NaHS
HG+Pal+NAC
HG+Pal+NaHS+AICAR

Figure.S5

A

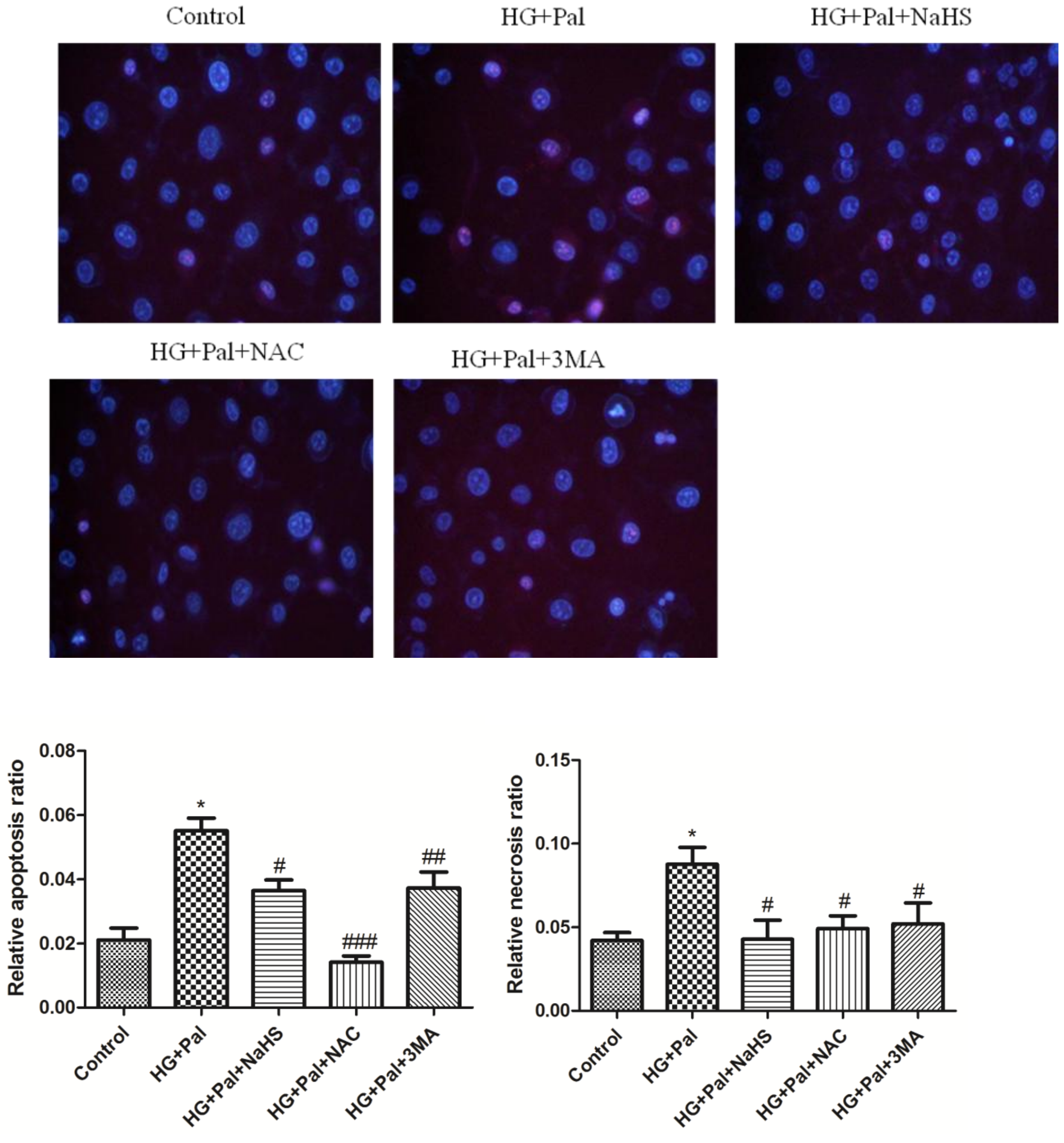
Control
HG+Pal
HG+Pal+NaHS
HG+Pal+NAC
HG+Pal+3MA
Relative apoptosis ratio
0.08
0.06
0.04
0.02
0.00
*
#
###
##
Control
HG+Pal
HG+Pal+NaHS
HG+Pal+NAC
HG+Pal+3MA
Relative necrosis ratio
0.15
0.10
0.05
0.00
*
#
#
#
Control
HG+Pal
HG+Pal+NaHS
HG+Pal+NAC
HG+Pal+3MA

B

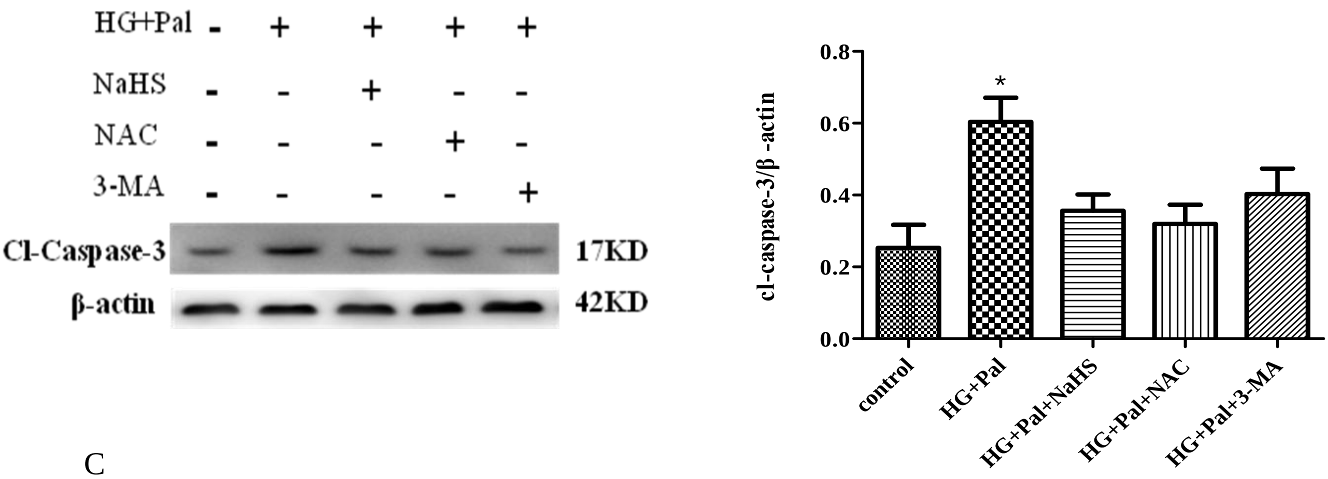
HG+Pal - + + + +
NaHS - - + - -
NAC - - - + -
3-MA - - - - +
Cl-Caspase-3
17KD
β-actin
42KD
cl-caspase-3/β -actin
0.8
0.6
0.4
0.2
0.0
*
control
HG+Pal
HG+Pal+NaHS
HG+Pal+NAC
HG+Pal+3-MA

C

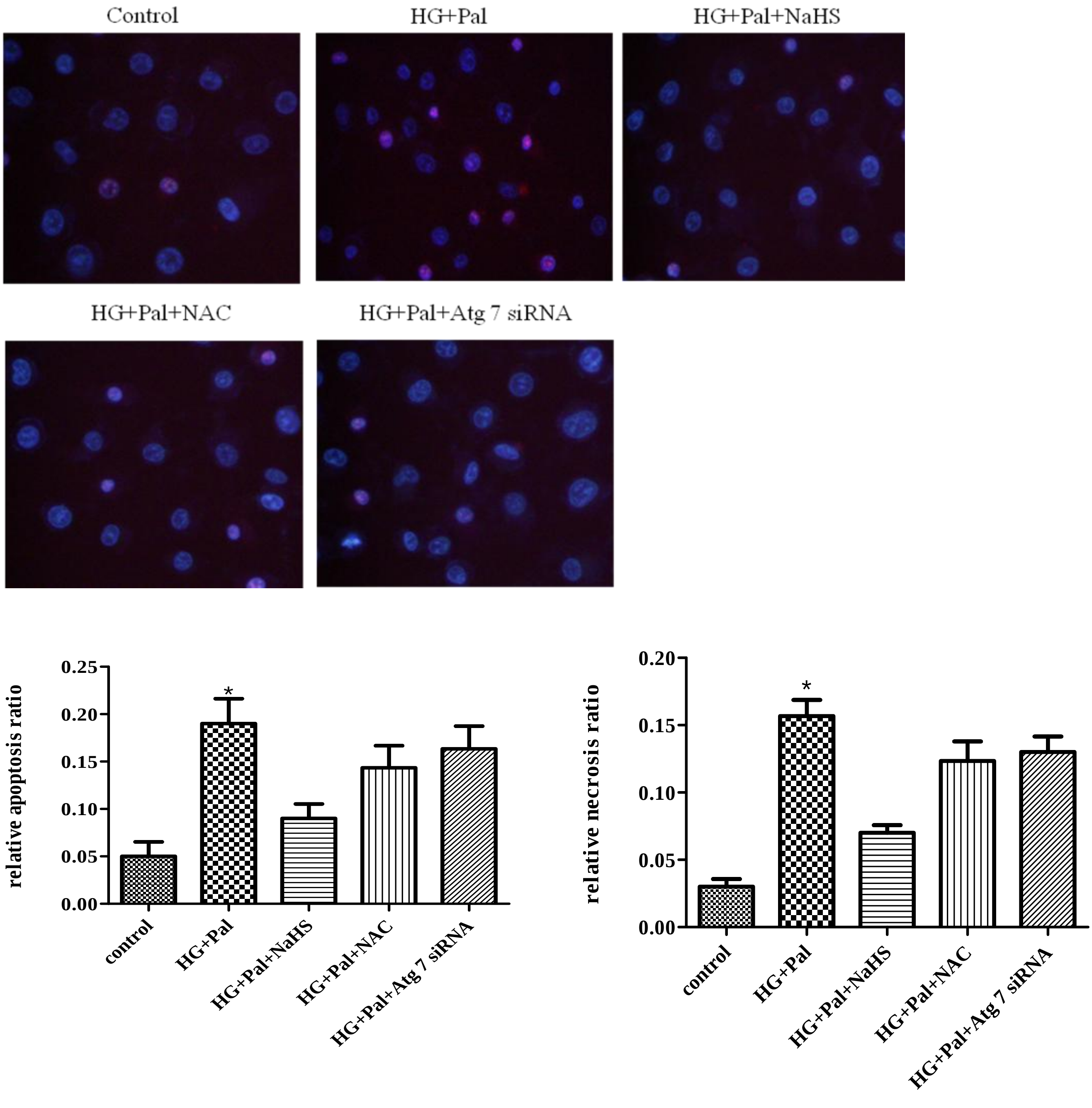
Control
HG+Pal
HG+Pal+NaHS
HG+Pal+NAC
HG+Pal+Atg 7 siRNA
relative apoptosis ratio
0.25
0.20
0.15
0.10
0.05
0.00
*
control
HG+Pal
HG+Pal+NaHS
HG+Pal+NAC
HG+Pal+Atg 7 siRNA
relative necrosis ratio
0.20
0.15
0.10
0.05
0.00
*
control
HG+Pal
HG+Pal+NaHS
HG+Pal+NAC
HG+Pal+Atg 7 siRNA
